# Approaches to multidrug-resistant organism prevention and control in long-term care facilities for older people: a systematic review and meta-analysis

**DOI:** 10.1186/s13756-021-01044-0

**Published:** 2022-01-15

**Authors:** Valerie Wing Yu Wong, Ying Huang, Wan In Wei, Samuel Yeung Shan Wong, Kin On Kwok

**Affiliations:** 1 JC School of Public Health and Primary Care, The Chinese University of Hong Kong, Room 419, 4/F, JC School of Public Health and Primary Care Building, Prince of Wales Hospital, Shatin, N.T., Hong Kong Special Administrative Region China; 2 Stanley Ho Centre for Emerging Infectious Diseases, The Chinese University of Hong Kong, Shatin, N.T., Hong Kong Special Administrative Region China; 3 Shenzhen Research Institute of The Chinese University of Hong Kong, Shenzhen, China; 4 Hong Kong Institute of Asia-Pacific Studies, The Chinese University of Hong Kong, Shatin, N.T., Hong Kong Special Administrative Region China

**Keywords:** Antimicrobial resistant, Antibiotic resistant, Multidrug-resistant, Methicillin resistant, Infection control, Infection prevention, Barrier precautions, Contact precautions, Nursing homes, Long-term care

## Abstract

**Background:**

Despite clear evidence of benefits in acute-care hospitals, controversy over the effectiveness of IPC measures for MDROs is perceptible and evidence-based practice has not been established.

**Objective:**

To investigate the effects of IPC interventions on MDRO colonization and infections in LTCFs.

**Data sources:**

Ovid MEDLINE, EMBASE, and CINAHL from inception to September 2020.

**Eligibility criteria:**

Original and peer-reviewed articles examining the post-intervention effects on MDRO colonization and infections in LTCFs.

**Interventions:**

(i) Horizontal interventions: administrative engagement, barrier precautions, education, environmental cleaning, hand hygiene, performance improvement, and source control; and (ii) vertical intervention: active surveillance plus decolonization.

**Study appraisal and synthesis:**

We employed a random-effects meta-analysis to estimate the pooled risk ratios (pRRs) for methicillin-resistant Staphylococcus aureus (MRSA) colonization by intervention duration; and conducted subgroup analyses on different intervention components. Study quality was assessed using Cochrane risk of bias tools.

**Results:**

Of 3877 studies identified, 19 were eligible for inclusion (eight randomized controlled trials (RCTs)). Studies reported outcomes associated with MRSA (15 studies), vancomycin-resistant Enterococci (VRE) (four studies), Clostridium difficile (two studies), and Gram-negative bacteria (GNB) (two studies). Eleven studies were included in the meta-analysis. The pRRs were close to unity regardless of intervention duration (long: RR 0.81 [95% CI 0.60–1.10]; medium: RR 0.81 [95% CI 0.25–2.68]; short: RR 0.95 [95% CI 0.53–1.69]). Vertical interventions in studies with a small sample size showed significant reductions in MRSA colonization while horizontal interventions did not. All studies involving active administrative engagement reported reductions. The risk of bias was high in all but two studies.

**Conclusions:**

Our meta-analysis did not show any beneficial effects from IPC interventions on MRSA reductions in LTCFs. Our findings highlight that the effectiveness of interventions in these facilities is likely conditional on resource availability—particularly decolonization and barrier precautions, due to their potential adverse events and uncertain effectiveness. Hence, administrative engagement is crucial for all effective IPC programmes. LTCFs should consider a pragmatic approach to reinforce standard precautions as routine practice and implement barrier precautions and decolonization to outbreak responses only.

**Supplementary Information:**

The online version contains supplementary material available at 10.1186/s13756-021-01044-0.

## Introduction

The emergence of multidrug-resistant organisms (MDRO) is a major public health concern in the twenty-first century [1]. It limits the effective antimicrobial treatment options for infections and increases the morbidity, mortality, and health care costs in health care settings worldwide [2–4].

Long-term care facilities for older people (short for “LTCFs”) play an important and unique role in MDRO transmission. They have long been regarded as reservoirs for antimicrobial resistance (AMR). Prevalence studies reported approximately three out of ten residents were colonized with methicillin-resistant Staphylococcus aureus (MRSA) [5–7]. Risk factors for MDRO colonization and acquisition have been well characterized [8]. The high colonization prevalence can be attributable to (i) a homelike environment where residents assemble in close proximity, frequently sharing recreation and dining areas, increases the risk of MRSA acquisition [9, 10] and (ii) the clustering of vulnerable individuals who are often older, have chronic illnesses, require indwelling or invasive devices and antibiotic use for sustained nursing care [11]. These high-risk individuals have frequent contact with healthcare workers [12]. This contact pattern offers opportunities for onward transmission and acquisition of MDROs, which facilitates intra- and inter-facility transmission [13, 14].

Although research and development of new antibiotics are considered the most direct approach to combat MDROs, financial and technical challenges, such as low profitability yield and lengthy clinical testing may hinder the process [15]. Evidence suggests a significant association between levels of antibiotic consumption and the incidence of antibiotic resistance at both the individual and community levels [16, 17]. As a result, the use of antibiotics alone is not a sustainable solution to avert the current AMR crisis.

Infection prevention and control (IPC) provides an alternative and practical solution to reduce MDRO colonization and prevent harm caused by MDRO infections. This approach comprises two types of interventions: horizontal and vertical. Horizontal interventions aim to control the transmission of multiple pathogens simultaneously by implementing standardized practices, while vertical strategies target a reduction in the transmission of specific pathogens with active screening programmes followed by decolonization [18]. National and local guidelines recommend IPC interventions to control MDRO transmission in LTCFs [19–21]. Both strategies are predominately adopted from acute-care settings or based on a consensus from experts. Recent narrative reviews have described the types of interventions without summarizing the data [22–24]. Studies quantifying intervention effects on MDROs are mostly conducted in acute-care hospitals, which have different contact patterns from LTCFs [25, 26]. A 2013 Cochrane review attempted to assess the effects of IPC interventions on MDRO transmission in LTCFs [27], but identified only one clustered randomized controlled trial (RCT), and hence could not provide any pooled effect estimates [28]. The RCT reported that interventions improved staff compliance to IPC practice but had no effect on MRSA prevalence among residents. Two other systematic reviews showed contrasting findings in reducing general infection rates in LTCFs [29, 30]. Lee et al. supported the effectiveness of behavioral change strategies using education, monitoring, and feedback with 17 studies [29], while Uchida’s team reported no effect of interventions from 24 articles [30]. Although the number of studies has increased over the years, the evidence has not been updated and remains inconclusive.

In light of this, our systematic review aims to renew the existing evidence and quantify the effects of IPC interventions on MDRO transmission in terms of reductions in colonization and associated infections in LTCFs, and to evaluate the quality of the current evidence.

## Methods

### Search strategies and selection criteria

We searched for studies published from inception to September 2020 with the following electronic databases: “Ovid MEDLINE”, “Ovid MEDLINE Epub ahead of print”, In-Process & Other Non-Indexed Citations”, “EMBASE”, and “CINAHL”. A combination of search terms encompassing four domains was developed: MDROs, LTCFs, IPC interventions, and colonization or infections (see Additional file 1). This review conforms to the Preferred Reporting Items for Systematic Reviews and Meta-Analyses (PRISMA) guidelines [31]. We include original, peer-reviewed articles that evaluated IPC interventions in LTCFs. Studies assessing IPC interventions during an outbreak (for example, outbreak reports) were excluded. We also excluded qualitative studies, conference papers, posters, commentaries, and review articles.

This review focuses on prevalent and clinically concerned MDROs in LTCFs: MRSA, vancomycin-resistant Enterococci (VRE), multidrug-resistant Gram-negative bacteria (MDR-GNB), those producing extended-spectrum beta-lactamases, and others that are resistant to multiple classes of antimicrobial agents (i.e., carbapenem-resistant Enterobacteriaceae (CRE), carbapenemase-producing Enterobacteriaceae, and Clostridium difficile (C.diff.)) [32].

We defined an LTCF as a public or private residential institution that primarily provides a high level of long-term personal and nursing care assistance to individuals who cannot live independently. Our review included studies specific to LTCFs for older people, but excluded those facilities that provided specialized nursing care to other populations or adopted different care models from LTCFs [33].

We categorized eight IPC interventions into either horizontal or vertical groups. Horizontal interventions included: (i) administrative engagement, (ii) barrier precautions, (iii) education, (iv) environmental cleaning, (v) hand hygiene, (vi) performance improvement, and (vii) source control; vertical interventions included decolonization of colonized subjects only. We described each IPC intervention in detail in an additional table (see Additional file 2).

The primary outcome was MRSA colonization amongst residents in our study settings, where colonization referred to bacteria multiplication in the body without causing any infection. The microbiological assessment indicated the culture positivity in specimens from colonized individuals. We counted multiple positive cultures from the same resident as one colonization episode. “Acquisition” was interpreted as synonymous with “colonization”. The secondary outcomes were colonization from other MDROs and all MDRO infections.

### Data extraction and quality assessment

Two authors (VW, YH) independently extracted data and assessed study quality. They identified the articles and subsequently screened them through the full-text papers. Three categories of data were extracted: study characteristics, methodologies, and measured outcomes. The reference lists of relevant review articles from the search were screened for additional studies. We excluded any extended studies in the meta-analysis to avoid duplicate data.

The study quality was assessed using the revised Cochrane risk-of-bias tool (ROB2) for RCTs [34], and the Cochrane Risk of Bias In Non-randomized Studies of Interventions (ROBINS-I) tool for non-randomized studies [35]. The risk of bias of each study was graded as high, with some concerns, and low in ROBs. Similarly, it was graded as serious, moderate, and low in ROBINS-I. The risk-of-bias visualization tool synthesized the assessment and presented the data in a plot [36].

Any disagreements in data extraction and quality assessment were resolved by consensus between VW and YH or through consultation with a third reviewer (KOK).

### Statistical analysis

Meta-analyses were performed using a random-effects model to estimate the pooled risk ratios (pRRs) with 95% confidence intervals (CIs). Meta-analyses on MRSA colonization based on 11 articles were conducted. We are limited to perform narrative syntheses on other outcomes due to three studies addressing the outcomes of interest and inconsistent outcome definitions or assessment methods for pooled analysis. In addition to visual inspection of forest plots, we quantified the heterogeneity across studies using the I^2^-statistics. Contour-enhanced funnel plot and Egger’s test were used to evaluate the publication bias—particularly small-study effects [37, 38]. However, the tests were not conducted for subgroup meta-analyses with fewer than ten studies, as the power of the tests would be too low to differentiate chance from real asymmetry [39].

Previous studies suggested that the effects of interventions varied by intervention duration [40, 41]*.* Subgroup analyses by short- (less than five months), medium- (6–11 months), and long- (12 months or longer) duration were conducted. We further summarized pRRs by different combinations of interventions for studies reporting a long duration. Finally, post hoc sensitivity analyses were performed only on studies with concurrent control to assess the robustness of the results.

The package “metaphor” of R Studio version 4.0.2 was used to perform the meta-analyses and subgroup statistical analyses [42, 43]. A p-value of less than 0.05 was considered statistically significant.

## Results

### Record retrieval

**Fig. 1:**
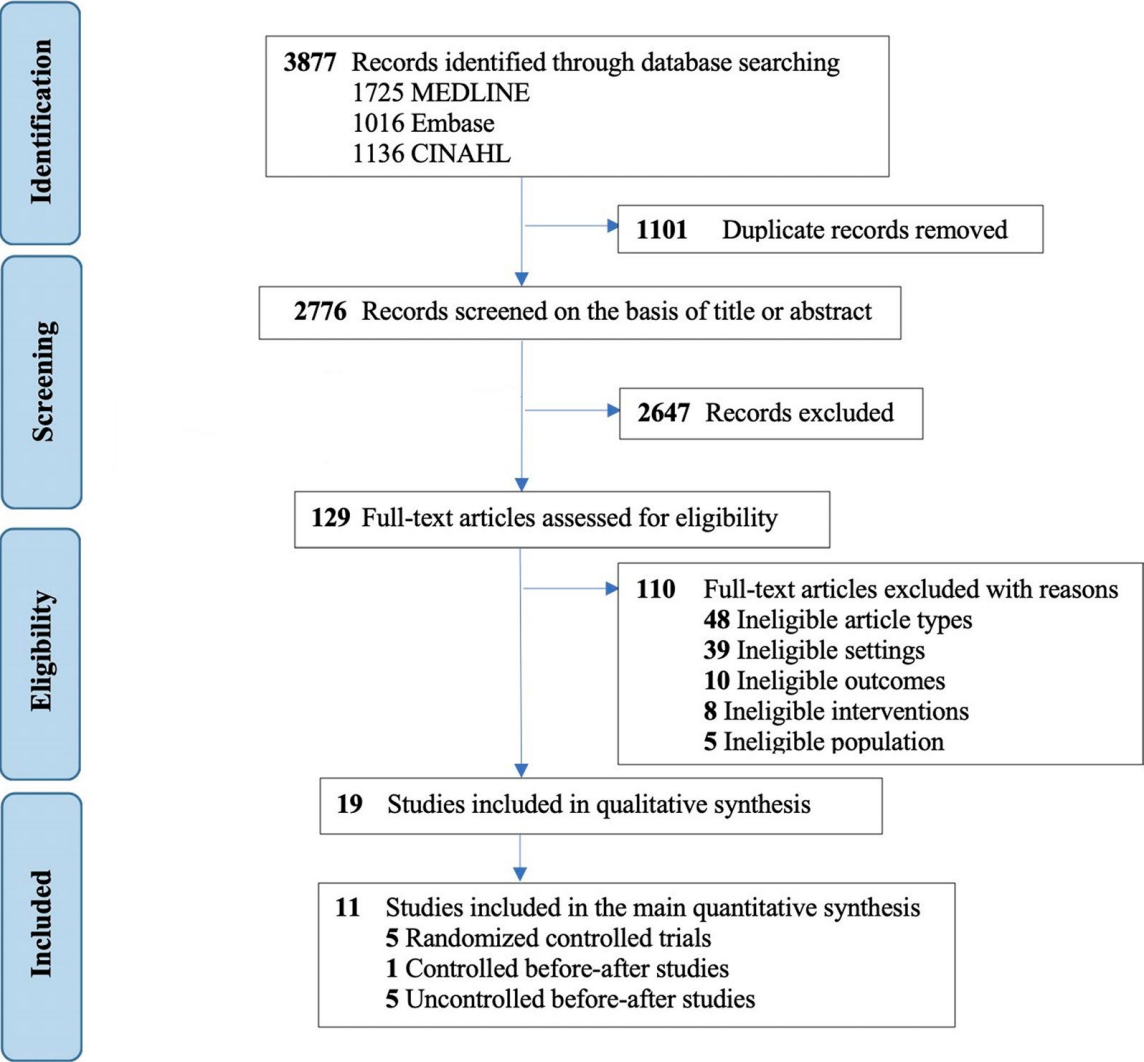
PRISMA flow diagram

The search strategy identified 3877 articles. Following the removal of duplicates, 2776 articles remained. After screening titles and abstracts, 129 articles were included for full-text review (Fig. 1). Nineteen articles met the inclusion criteria and were included in the systematic review [28, 44–61].

### Characteristics of included studies

Among the 19 articles included, there were eight RCTs [28, 44, 47–49, 53, 56, 57], eight uncontrolled before-after studies [45, 46, 50–52, 55, 59, 60], one controlled before-after study [54], and two interrupted time-series studies [58, 61]. One article was an extended study of another [44, 48]. Two articles were based on the same study but presented different outcomes [56, 57]. Fifteen studies associated with MRSA [28, 44, 46–54, 56, 57, 60, 61], four with VRE [53, 55, 58, 61], two with C.diff. [58, 59], and two with GNB [45, 53]. Seventeen studies evaluated multifaceted interventions [28, 44–51, 53, 55–61], and two employed a single-component intervention [52, 54]. Twelve studies were conducted in the United States [46, 51–61], four in Europe [28, 44, 48, 50], two in Asia [47, 49], and one in the Middle East [45] (Table 1).

**Table 1:** Characteristics of included studies

References	Country	Study design	Control type	MDRO type	Measured outcomes	Staff compliance measured	Interventions (duration, months)	No. of residents analyzed (baseline)	Summary findings
Baldwin et al. [28]	Northern Ireland	Clustered RCT	Concurrent	MRSA	Colonization	Y	ED + PI (3, 6, 12)	793	No effect
Bellini et al. [44]	Switzerland	Clustered RCT	Concurrent	MRSA	Colonization; infections	N	DC + EC + ED (12)	4750	No effect^1^
Ben-David et al. [45]	Israel	Uncontrolled before-after	Historical	CRE	Acquisition	N	AE + BP + ED + PI (84)	~ 20000^2^	Reduction^3^
Bowler et al. [46]	United States	Uncontrolled before-after	Historical	MRSA	Colonization	N	DC + EC + ED (13)	687	Reduction^3^
Chuang et al. [47]	Hong Kong SAR, China	Clustered RCT	Concurrent	MRSA	Colonization	Y	BP + EC + ED + HH + PI (6, 9, 12, 15)	2776	No effect
Hequet et al.^4^ [48]	Switzerland	Clustered RCT	Concurrent	MRSA	Colonization	N	DC + EC + ED (12, 60)	NS	No effect
Ho et al. [49]	Hong Kong SAR, China	Clustered RCT	Concurrent	MRSA	Infections	Y	AE + ED + HH + PI (4)	2407	Reduction^3^
Horner et al. [50]	United Kingdom	Uncontrolled before-after	Historical	MRSA	Colonization	Y	ED + PI (6, 9, 12, 18, 24)	2237^5^	No effect
Jaqua-Stewart et al. [51]	United States	Uncontrolled before-after	Historical	MRSA	Colonization; infections	N	BP + DC + ED + SC (12, 39)	42	Reduction^3^
Kauffman et al. [52]	United States	Uncontrolled before-after	Historical	MRSA	Colonization	N	DC (7^6^, 5^7^)^8^	321	Reduction^3,9^
Mody et al. [53]	United States	Clustered RCT	Concurrent	MRSA; VRE; GNB	Colonization; infections	Y^10^	BP + ED + HH + PI (24)	418	Reduction^3^
Morgan et al. [54]	United States	Controlled before-after	Concurrent	MRSA	Acquisition; infections	N	BP (48)	75,414	No effect
Ostrowsky et al. [55]	United States	Uncontrolled before-after	Historical	VRE	Colonization	Y^11^	BP + EC + ED + HH (12, 24)	5221	Reduction^3^
Peterson et al.^12^ [56]	United States	Clustered RCT	Concurrent	MRSA	MRSA infections	N	DC + EC + ED + SC (12, 24)	7069	Reduction^3^
Schora et al. [57]	United States	Clustered RCT	Concurrent	MRSA	Colonization	N	DC + EC + ED + SC (12, 24)	4424	Reduction^3^
Schweon et al. [61]	United States	Uncontrolled interrupted time series	Historical	MRSA; VRE; C.diff	Infections	Y	AE + ED + HH + PI (22)	NS	No effect
Silverblatt et al. [58]	United States	Uncontrolled interrupted time series	Historical	VRE	Colonization	N	BP + DC + ED + HH (28)	NS	Absence of outcome
Singh et al. [59]	United States	Uncontrolled before-after	Historical	C.diff	C.diff infections	N	AE + BP + EC + ED + HH (33)	~ 9381^13^	Reduction^3^
Thomas et al. [60]	United States	Uncontrolled before-after	Historical	MRSA	Colonization; MRSA infections	N	BP + ED (3)	164	Reduction^3^

AE, administrative engagement; BP, barrier precautions; BSI, bloodstream infection; C.diff., Clostridium difficile; DC, decolonization; ED, education; EC, environmental cleaning; GNB, Gram-negative bacteria; HH, hand hygiene; MRSA, methicillin-resistant Staphylococcus aureus; PI, performance improvement; NS, not specified; SC, source control; UC, usual care; VRE, vancomycin-resistant enterococci

^1^Colonization prevalence declined in both groups. Absence of invasive infections in both groups

^2^The sample size was not specified. We reported the number of beds in nursing homes

^3^Statistically significant reduction

^4^Extended study from Bellini et al. [44]

^5^Since the information was insufficient in survey two and three, we only included participants from survey one and four

^6^In Phase one, residents were decolonized in their anterior nares only for seven months

^7^In Phase two, residents were decolonized in both their anterior nares and wound for five months

^8^We only included results from Phase one in the quantitative analysis since the results from Phase two might be affected by the carryover effect

^9^Reductions were reported when decolonization was applied in both nares and wounds but not nares only

^10^A structured 30-min observations monitored HCW activities and their use of barrier precautions, but results were not reported

^11^The authors used self-reported questionnaires to monitor the staff compliance before and after the interventions implemented

^12^Schora et al. [57] and Peterson et al. [56] are based on the same study with the same study sample

^13^The authors reported there were a total of 9,288,098 resident days. We estimated there were around 9381 residents after dividing the number of resident days with 30 days and 33 months

### Effectiveness of IPC interventions on MRSA colonization

**Fig. 2:**
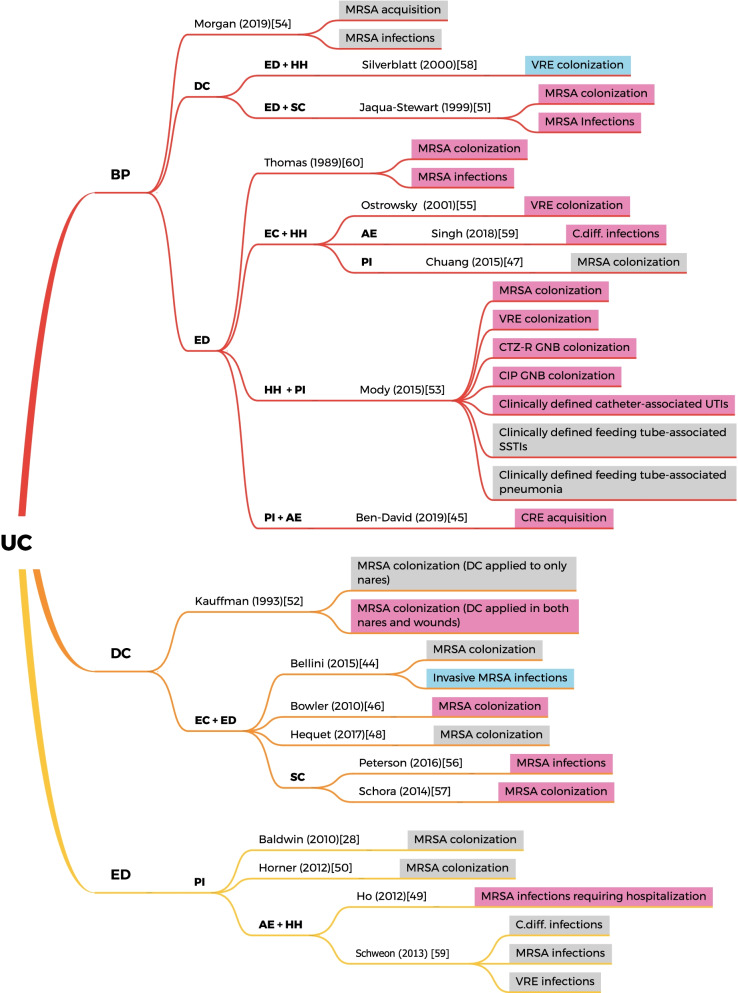
Components and outcomes of included studies. (Pink—reductions in outcome reported by authors; Grey—no effect in outcome reported by author—Blue, absence of outcome)

Of the 12 studies on MRSA colonization, the directions of intervention effects were divided: half of the studies found insignificant or no effects [28, 44, 47, 48, 50, 54], while the other half reported significant reductions [46, 51–53, 57, 60]. Seven studies involved at least three intervention components [44, 46–48, 51, 53, 57]. The most common interventions were education (83%) [28, 44, 46–48, 50, 51, 53, 57, 60], decolonization (50%) [44, 46, 48, 51, 52, 57], and environmental cleaning (42%) [44, 46–48, 57] (Fig. 2). The interventions included in each study are summarized in a table (see Additional file 3). Two studies evaluated the individual effect of barrier precautions and decolonization. One study showed that barrier precautions alone did not affect MRSA acquisition after controlling for patient demographics, comorbidity, and year of admission (odds ratio, 0.97 [95% CI 0.85–1.12]; p = 0.71) [54], while the other reported decolonizing both the nasal cavities (nares) and wounds yielded a reduction in mean monthly colonization rate from 22.7% to 11.5% (p = 0.0001) [52]. Only four studies reported change in compliance followed by the interventions: three on hand hygiene [28, 47, 50], and one on barrier precautions [53]. However, the evaluation methods were not standardized (see Additional file 4).

**Fig. 3:**
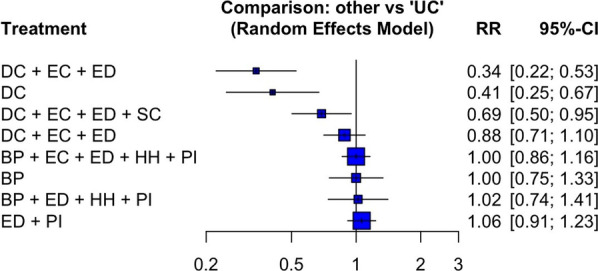
Forest plot for studies evaluating the long-term intervention effects on MRSA colonization by components. (“DC + EC”, Decolonization and environmental cleaning; “DC”, Decolonization only; “DC + EC + ED + SC”, Decolonization, environmental cleaning, education, and source control; “DC + EC + ED”, Decolonization, environmental cleaning, and education; “BP + EC + ED + HH + PI”, Barrier precautions, environmental cleaning, education, hand hygiene, and performance improvement; “BP”, Barrier precautions; “BP + ED + HH + PI”, Barrier precautions, education, hand hygiene, and performance improvement; “ED + PI”, Education and performance improvement; “UC”, Usual care)

We excluded Hequet et al. (2017) since it is a follow-up study from Bellini et al. (2015) [44, 48]. Our meta-analysis of 11 articles showed that IPC interventions were not associated with the reduction regardless of the intervention duration (long: pRR 0.81 [95% CI 0.60–1.10]; medium: pRR 0.81 [95% CI 0.25–2.68]; short: pRR 0.95 [95% CI 0.53–1.69]) [28, 44, 46, 47, 50–54, 57, 60]. We present the forest plot for studies evaluating MRSA colonization in an additional figure (see Additional file 5). Neither single-component nor multifaceted interventions resulted in a larger decrease in MRSA colonization (Fig. 3). While active surveillance and decolonization reported reductions, albeit statistically insignificant in larger studies, in colonization (range: pRR 0.34 [95% CI 0.22–0.53] to 0.88 [95% CI 0.71–1.10]), interventions involving mainly barrier precautions (range: pRR 1.00 [95% CI 0.75–1.33] to 1.02 [95% CI 0.74–1.41]) and education (pRR 1.06 [95% CI 0.91–1.23]) had no effects on MRSA colonization (Fig. 3). Our findings did not change when the analyses restricted only to studies with concurrent control (long: pRR 0.94 [95% CI 0.83–1.07]; medium: pRR 1.01 [95% CI 0.10–10.21]; short: RR 0.96 [95% CI 0.73–1.26]). The sensitivity analysis demonstrated a significant drop in the I^2^ of long-term interventions to 1% (p = 0.41); the I^2^ of medium-term interventions reduced only slightly to 77% (p = 0.04); and, the I^2^ of short-term interventions remained unchanged at 0%. The forest plot for sensitivity analysis is presented as an additional figure (see Additional file 6).

### Effectiveness of IPC interventions on other outcomes

Three studies employing altogether barrier precautions, hand hygiene, and education reported either reduced VRE colonization or free from new acquisition [53, 55, 58]. Of the two studies reporting reductions, one reported a cluster- and covariate-adjusted hazard ratio (HR) of 0.85 [95% CI 0.45–1.60] (p = 0.61) [53], while another found a relative risk of 0.30 [95% CI 0.20–0.70] (p = 0.001) [55]. Two studies which included barrier precautions, education, and performance improvement in their IPC programme found decreases in GNB acquisition. One of them reporting administrative engagement decreased CRE acquisition from 0.5 per 10 000 patient-days at baseline to 0.3 per 10 000 patient-days two years after implementation [45]. The other study which also included hand hygiene reported an insignificant cluster- and covariate-adjusted HR of 0.90 [95% CI 0.60–1.33] (p = 0.59) [53]. There were nine studies evaluating the effects of IPC interventions on infections, of which six reported reduction [49, 51, 53, 56, 59, 60], one had no infection episodes [44], and two found no effects [54, 61]. The six studies reporting reductions in infections summarized their results in various metrics with two reporting significant rate ratios ranged from 0.54 [95% CI 0.30–0.97] to 0.61 [95% CI 0.38–0.97] (p = 0.04) [49, 53] and four showing relative reductions in infection rates ranging from 25.9% to 99.7% [51, 56, 59, 60]. The rationale for meta-analyses in other outcomes was limited due to heterogeneity in study designs and lack of studies.

### Effectiveness of IPC interventions on MDRO outcomes involving administrative engagement

All studies actively involving the administrations reported reductions in either colonization or infections [45, 49, 59, 61], although one did not reach 5% significant level [61]. Among the studies that did not result in outcome reductions, both alluded their failures to lack of organizational commitment [28, 47]*.*

### Study quality

**Fig. 4:**
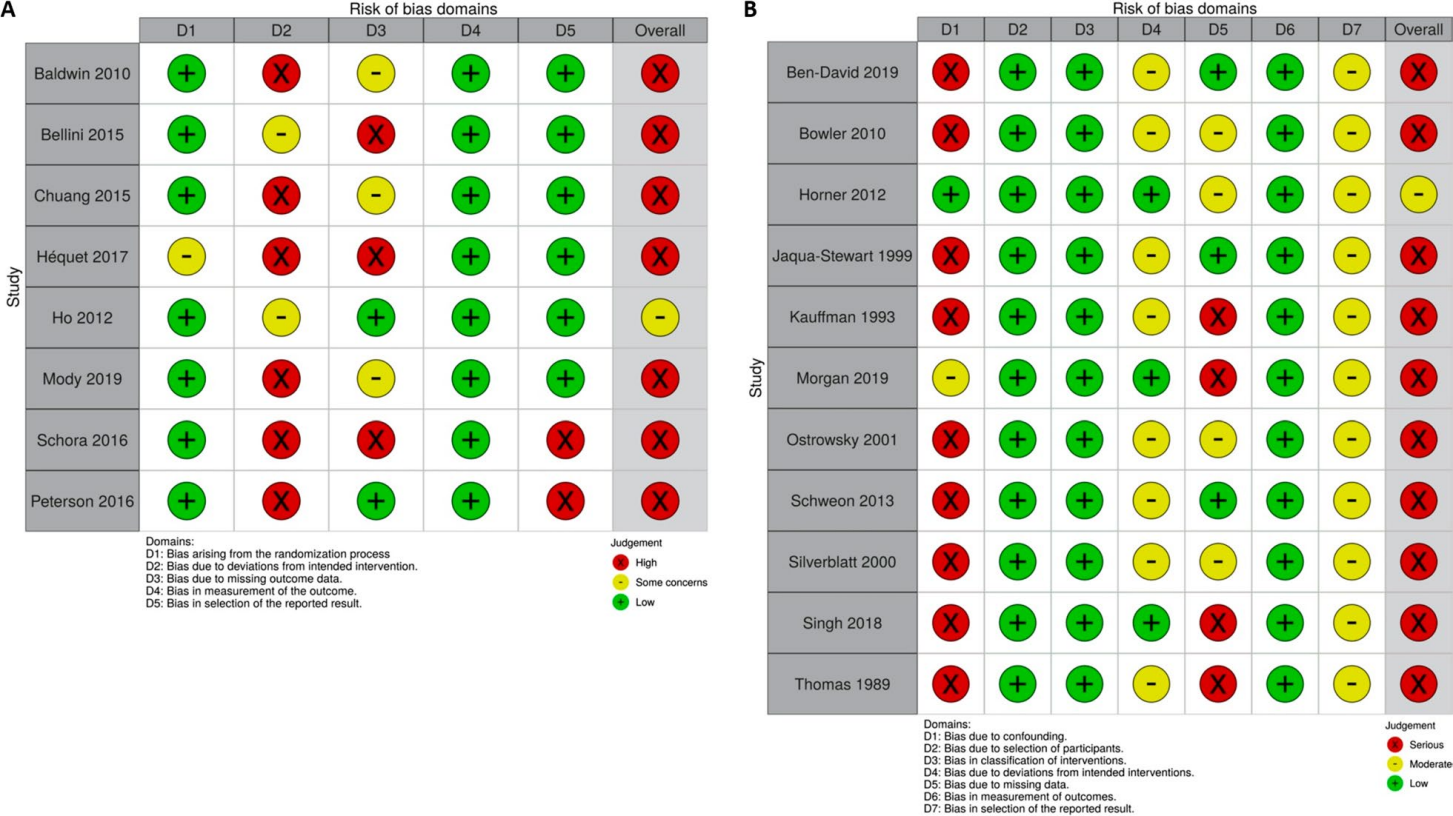
**a** Risk of bias plot for randomized controlled trials. **b** Risk of bias plot for non-randomized studies

The risk of bias among 19 studies was generally high (Fig. 4a, b). Of the eight RCTs [28, 44, 47–49, 53, 56, 57], all but one were at high risk of bias [49]. The only one with “some concerns” of bias reported significant reductions in MRSA infections requiring hospitalization following the implementation of a multimodal strategy [49]. The main reason for downgrading was that most studies did not appropriately adjust for the imbalance of missing outcome data in the analytical stage. The risk of bias assessment using ROB2 tool is presented in an additional table (see Additional file 7). Similarly, we rated the risk of bias of one non-RCTs as “moderate” [50], while others were as “serious” [45, 46, 51, 52, 54, 55, 58–61]. Unadjusted confounders without randomization were the main cause of degradation. The risk of bias assessment using ROBINS-I is available (see Additional file 8). The controlled before-after study with a moderate-level risk of bias reported a small but insignificant increase in MRSA colonization after the implementation of an IPC programme [50].

### Publication bias and small study effects

The funnel plot suggests differences in intervention tendency effects among smaller and larger studies (see Additional file 9). Results from the majority of moderate-sized studies were statistically insignificant but that among studies with a small sample size was generally significant. Small-sized studies with low methodological quality produced exaggerated positive intervention effect estimates. Egger’s test also supports the presence of small-study effects (intercept = − 4.05; p = 0.03).

## Discussion

### Principal findings

This systematic review provides an up-to-date, critically appraised, and comprehensive literature syntheses of IPC interventions’ effect on MDRO risk in LTCFs. Evidence was overall low in quality. Our meta-analysis did not demonstrate a significant decrease in MRSA colonization following IPC interventions. While the pooled estimates varied by intervention types, none consistently produced significant results of which vertical interventions may reduce MRSA colonization but horizontal interventions had no effects. Some smaller studies with low methodological quality produced exaggerated positive intervention effect estimates. IPC interventions may reduce VRE and GNB colonization while they had an inconsistent impact on MDRO infections. Notably, administrative engagement is a core component in all successful IPC programmes to curtail MDRO colonization in LTCFs.

### Comparison with previous literature

This review adds to the existing knowledge in several ways.

First, this review did not find compelling evidence to support the effectiveness of IPC interventions on MRSA reduction, in particular barrier precautions. Although expert guidance recommends the use of screening and barrier precautions for patients colonized or infected with MDROs, [32, 62, 63], a rising number of studies have questioned their effectiveness and benefits on MDRO control in different health care settings. A large cluster-randomized trial conducted in an intensive care unit found the use of active screening and barrier precautions has been ineffective in reducing transmission of MRSA and VRE [64]. An intervention study in the United States found barrier precautions had no impact on MRSA acquisition and infections [54]. Discontinuing barrier precautions was also not associated with the infection rates surge in a recent meta-analysis [65]. The actual effectiveness of barrier precautions is likely to be conditional on resource availability. In an ideal condition with unlimited resources, i.e., low staff-to-resident ratios, implementing barrier precautions may not necessarily compromise the use of personal protective equipment. However, in low-resource settings like LTCFs, the heavy clinical workload is a major barrier to infection control [66]. In a prospective cohort study, one thousand and thirteen observations were conducted on the adherence of isolation practices among healthcare workers as the burden of isolation increased (20% or less to greater than 60%), a substantial decrease in compliance with hand hygiene (from 43.6 to 4.9%) and with all five components of contact precautions [67].

While evidence has not yet confirmed the effectiveness, barrier precautions have also been associated with a number of adverse events. Adopting barrier precautions constrains residents’ mobility and thus results in social stigma and isolation [9]. A matched-controlled study found that isolation led to medical errors and patient neglect [68]. A systematic review summarized four main adverse outcomes related to barrier precautions: (i) less patient-health care worker contact time, (ii) delays in care and more non-infectious adverse events, (ii) increased patient depression and anxiety symptoms, and (iv) decreased patient satisfaction with care [69]. A survey revealed that isolation increased anxiety of both patients and family members [70]. Barrier precautions also impose burdens to health care workers, which compromises the compliance of hand hygiene and use of personal protective equipment, as the number of isolated patients increases [67]. Overall, the risks of barrier precautions to residents seemed to offset its benefits on MDRO control in LTCFs.

Second, in terms of vertical interventions, although the use of decolonization among patients at risk of infections for a short period was supported [71], its long-term use remains inconclusive. The temporary effect can be attributable to the high rate of recolonization, which is particularly relevant to this unique population in LTCFs. A pilot study conducted in an outpatient chronic haemodialysis unit and an RCT in two LTCFs found the mean recolonization time was 27 and 45 days, respectively [72, 73].

Apart from the temporary effect of decolonization, multiple colonization further complicates the effectiveness of decolonization therapy in LTCFs. Decolonizing a single organism may not be adequate in reducing the risk of transmission. A nested case–control study reported that residents colonized with multidrug-resistant Acinetobacter baumannii were more likely to be colonized with another type of GNB [74]. Besides, decolonization therapy can lead to adverse reactions and increased secondary drug resistance. Of the patients decolonized with mupirocin in an RCT, 25% developed gastrointestinal adverse reactions, and 5% progressed into a high level of drug resistance [75]. Therefore, the potential risk of decolonization therapy should be weighed alongside its benefits to MDRO control in LTCFs.

Third, the role of administration has proven to be crucial in various contexts. A few studies suggested the direct and indirect benefits of engaging administrative commitment. A semi-structured interview of 20 clinicians revealed how hospital administrative engagement facilitated interventions in reducing surgical-site infections [76]. Similarly, a Thai national survey described an association between good-to-excellent administrative support and high degree of adherence to environmental disinfection [77]. A cross-sectional survey reported that hospitals with more effective leadership showed better compliance in hand hygiene and were less likely to report implementation barriers [78]. The health belief model can be used to explain the association [79, 80]: as perceived barriers to action is one of the six key factors that influence health behaviours, a supportive administrative engagement not only eliminates the perceived barriers to implementation but also promotes learning, allocates adequate resources, and creates a facilitative and collaborative environment [81].

Fourth, studies have long demonstrated that interventions designed to improve health outcomes are more effective when implemented in bundles [82]. Over the past decade, multifaceted interventions in IPC have been reported to be more effective in reducing MDRO burden in LTCFs [29, 83], and have been widely adopted in national guidelines and recommendations [32, 84]. However, there might be a possible association between the number of interventions and the effectiveness of the programme—a simulation study found synergistic effects of combination interventions for MRSA control in nursing homes, assuming there were no resource constraints [85]. However, after taking cost into account, the more interventions simultaneously employed in the bundle did not correlate with a higher cost-effectiveness than an isolated daily cleaning intervention [86]. The challenge around resource constraints could explain why the number of components did not seem to affect the effectiveness of the programmes in our study.

### Strengths and limitations

This study and its evidence are affected by some inherent limitations. First, in the majority of the studies, very few data on adherence to IPC interventions was reported. However, the compliance of health care workers to interventions greatly influences the assessment of their effectiveness. A low level of adherence would potentially shrink the estimates of the intervention effects towards the null. However, poor compliance to IPC interventions, particularly hand hygiene, is a persistent problem in infection control practice. Although the degree of compliance could affect the reported effectiveness of the interventions, our results reflect and can be generalized to situations under the constraint of limited resources. Second, the multiplicity of outcome measures could limit the potential to synthesize results. Since most studies usually include a number of interventions, it is difficult to sort out the independent effects of a single intervention component. Third, there is also a potential for misclassification of interventions as most of the studies did not report the routine interventions as a part of the IPC bundles. Fourth, we limited our meta-analysis to all studies that reported MRSA colonization. However, this approach is prone to high heterogeneity across studies due to different outcome definitions, study designs, and intervention components. Although we have grouped interventions into eight categories and performed subgroup analyses to identify the source of heterogeneity, it is still difficult to draw a definitive conclusion. Fifth, we found only two moderate-quality studies, and they provided inconsistent evidence of effectiveness. The low quality of the study affects the internal validity of our review. Last, the possibility of spontaneous decolonization and recolonization could affect the estimate of individual studies, which may impact the estimates of this review. However, the spontaneous decolonization rate is low while recolonization is common in LTCFs. A recent study found that the spontaneous decolonization rate among intensive care unit patients colonized by ESBL-E was only 2.5% [87], while that of MRSA was 4.1% in LTCFs [88]. On the other hand, the recolonization rate of MRSA is high among nursing home residents. In a randomized, double-blind, placebo-controlled trial, 86% of decolonized nursing home residents became recolonized with the same pretherapy strain of MRSA at 90 days after study entry [73].

Despite its limitations, our review provides the first comprehensive evidence synthesis of the association between IPC interventions and MDRO colonization and infections in LTCFs. Infection prevention and control interventions in LTCFs play an important role in reducing the spread of MDROs across hospitals and community settings. Yet, significant gaps and inconsistencies in current practices are evident. Methodologically less rigorous studies tend to overestimate the effects of IPC programmes, which poses a high risk of bias on the current guidelines and hinders decision-making in public health policies. More rigorously controlled studies are required to answer several key questions: (i) What are the most effective interventions in reducing MDRO-related colonization and infections in LTCFs—in terms of their ideal composition (single-component or multifaceted)?; (ii) What are the institutional and infrastructural determinants for an effective IPC programme? For example, staff-to-resident ratios, bed occupancy, the density of hand washing/rubbing points, etc.?; (iii) What are the short- and long-term intervention adherence from the staff in LTCFs?; and (iv) How does the intervention adherence affect the effectiveness of programmes?

Till we have the answers to these questions, in the absence of a clear benefit from interventions and the presence of evidence that various adverse outcomes linked with decolonization and barrier precautions, basic standard precautions—which generally include hand hygiene, environmental cleaning, and staff education that are low-cost and not menacing to residents—remains the optimal approach in LTCFs. Since the success of a programme rests with high institutional compliance [89], reinforcing strict adherence should be the primary strategy to maximize the effects of standard precautions. An engaged administration with clearly defined goals promotes compliance through consultations with frontline staff and adequate resource allocation. It helps to identify and remove implementation barriers for routine standard precautions. Facility managers have to judiciously evaluate the benefits and risks of interventions that have potential negative impacts on residents. When the benefits to residents outweigh the risks, a detailed execution plan should be constructed, predominately when and on what criteria to discontinue the interventions. The possibility of not placing residents with these interventions should always be considered before other options.

## Conclusions

The proliferation of AMR limits treatment options for patients and is no longer adequately addressed solely by research and development of new antibiotics. Apart from curtailing inappropriate use of antibiotics, IPC interventions are rational steps in reducing the colonization and infection risks. However, existing evidence suggested IPC interventions without administrative engagement offer little effect on MDRO control in long-term care settings. Securing the administrative commitment, LTCFs can reinforce the standard precautions in routine care of residents and weigh carefully before applying barrier precautions and decolonization. Prospective studies can explore how strategies promoting prudent use of antibiotics, for instance, antimicrobial stewardship programmes and point-of-care testing, can work hand in hand with the refined IPC strategies to formulate a comprehensive programme in LTCFs.

## Supplementary Information

**Additional file 1.** Search strategies.

**Additional file 2.** Description of infection prevention and control strategies.

**Additional file 3.** Interventions included in each studies.

**Additional file 4.** Characteristics of included studies.

**Additional file 5.** Forest plot for studies evaluating MRSA colonization.

**Additional file 6.** Forest plot for sensitivity analysis.

**Additional file 7.** Risk of bias assessment for randomized trials using Cochrane risk-of-bias tool.

**Additional file 8.** Risk of bias assessment for non-randomized intervention studies using Cochrane risk of bias in non-randomized studies of intervention tool.

**Additional file 9.** Contour-enhanced funnel plot of studies.

## Data Availability

This review was based on data extracted from published papers available in the public domain.

## Abbreviations

AE: Administrative engagement
AMR: Antimicrobial resistance
BP: Barrier precautions
C.diff.: Clostridium difficile
CI: Confidence intervals
CIP-R: Ciprofloxacin-resistant
CRE: Carbapenem-resistant Enterobacteriaceae
CTZ-R: Ceftazidime-resistant
DC: Decolonization
EC: Environmental cleaning
ED: Education
GNB: Gram-negative bacteria
HH: Hand hygiene
HR: Hazard ratio
IPC: Infection prevention and control
KOK: Kin On Kwok
LTCF: Long-term care facility
MDR: Multidrug-resistant
MDRO: Multidrug-resistant organism
MRSA: Methicillin-resistant Staphylococcus aureus
P: P-value
PI: Performance improvement
PRISMA: Preferred reporting items for systematic reviews and meta-analyses
pRR: Pooled risk ratios
RCT: Randomized controlled trial
ROB2: Revised Cochrane risk-of-bias
ROBINS-I: Cochrane risk of bias in non-randomized studies of interventions
RR: Risk ratios
SC: Source control
SSTI: Skin and soft tissue infection
UC: Usual care
UTI: Urinary tract infection
VRE: Vancomycin-resistant Enterococci
VW: Valerie Wing Yu Wong
YH: Ying Huang
