# Supplementary material for: Approaches to multidrug-resistant organism prevention and control in long-term care facilities for older people: a systematic review and meta-analysis
Source: Antimicrob Resist Infect Control. 2022 Jan 15;11:7. doi: 10.1186/s13756-021-01044-0 (PMC8761316; doi:10.1186/s13756-021-01044-0)
Supplement: Supplementary file 1 — Additional file 1. Search strategies. [file 13756_2021_1044_MOESM1_ESM.docx]

**Additional file 1. Search strategies.**

| **Database** | **Search algorithm** | | |
| --- | --- | --- | --- |
| **Ovid Medline** | **Organisms**   1. multidrug-resistant organism*.ti,ab,kw. 2. mdro.ti,ab,kw. 3. antimicrobial resistan*.ti,ab,kw. 4. amr.ti,ab,kw. 5. extended spectrum beta-lactamase.ti,ab,kw. 6. esbl.ti,ab,kw. 7. methicillin-resistant staphylococcus aureus/ 8. methicillin-resistant staphylococcus aureus.ti,ab,kw. 9. methicillin resistance/ 10. methicillin resistance.ti,ab,kw. 11. mrsa.ti,ab,kw. 12. vancomycin-resistant enterococci/ 13. vancomycin-resistant enterococci.ti,ab,kw. 14. vancomycin resistance/ 15. vancomycin-resistant.ti,ab,kw. 16. vre.ti,ab,kw. 17. clostridium difficile/ 18. clostridium difficile.ti,ab,kw. 19. carbapenem-resistant enterobacteriaceae/ 20. carbapenem-resistant enterobacteriac*.ti,ab,kw. 21. cre.ti,ab,kw. 22. carbapenemase-producing enterobacteriac*.ti,ab,kw. 23. cpe.ti,ab,kw. 24. acinetobacter baumannii/ 25. acinetobacter baumannii.ti,ab,kw. 26. mdrab.ti,ab,kw. 27. mdr-ab.ti,ab,kw. 28. pseudomonas aeruginosa/ 29. pseudomonas aeruginosa.ti,ab,kw. 30. mdrpa.ti,ab,kw. 31. mdr-pa.ti,ab,kw. 32. klebsiella pneumoniae/ 33. klebsiella pneumoniae.ti,ab,kw.   **Long-term care facilities**   1. homes for the aged/ 2. nursing home/ 3. long term.ti,ab,kw. 4. Day Care, Medical/ 5. (elderly adj2 (home* or cent* or facilit* or unit* or institution*)).ti,ab,kw. 6. (geriatric adj2 (home* or cent* or facilit* or unit* or institution*)).ti,ab,kw. 7. (residential adj2 (home* or cent* or facilit* or unit* or institution*)).ti,ab,kw. 8. (retirement adj2 (home* or cent* or facilit* or unit* or institution*)).ti,ab,kw.   **Interventions**   1. infection control/ 2. infection control.ti,ab,kw. 3. standard care.ti,ab,kw. 4. personnel, hospital/ or hand disinfection/ or hand hygiene/ or hygiene/ or anti-infective agents, local/ 5. hand hygiene.ti,ab,kw. 6. contact precaution.ti,ab,kw. 7. antimicrobial stewardship/ 8. antimicrobial stewardship.ti,ab,kw. 9. disinfection/ 10. environment* clean*.ti,ab,kw. 11. decoloni#ation.ti,ab,kw. 12. decontamination/ 13. decontamination.ti,ab,kw. 14. equipment contamination/ 15. sanitation/ 16. patient isolation/ or social isolation/ 17. isolation.ti,ab,kw. 18. quarantine/ 19. quarantine.ti,ab,kw. 20. source control.ti,ab,kw. 21. mass screening/ 22. surveillance.ti,ab,kw. 23. screening.ti,ab,kw. 24. notification.ti,ab,kw. 25. cohorting.ti,ab,kw. 26. chlorhexidine.ti,ab,kw. 27. mandatory reporting.ti,ab,kw. 28. hand rub.ti,ab,kw. 29. glove*.ti,ab,kw.   **Outcomes of interest**   1. burden.ti,ab,kw. 2. mortality.ti,kw. 3. death*.ti,kw. 4. "costs and cost analysis"/ 5. cost*.ti,kw. 6. hospitali#ation.ti,kw. 7. length of stay.ti,kw. 8. productiv*.ti,kw. 9. morbidity.ti,kw. 10. coloni#ation.ti,ab,kw. 11. infection.ti,ab,kw. 12. acquisition.ti,ab,kw. 13. incidence/ 14. incidence.ti,ab,kw. 15. prevalence/ 16. prevalence.ti,ab,kw.   #87. OR (#1 to #33)  #88. OR (#34 to #41)  #89. OR (#42 to #70)  #90. OR (#71 to #86)  #91. AND (#87 to #90) | | |
| **EMBASE** | **Organisms**   1. multidrug-resistant organism*.ti,ab,kw. 2. mdro.ti,ab,kw. 3. antimicrobial resistan*.ti,ab,kw. 4. amr.ti,ab,kw. 5. extended spectrum beta-lactamase.ti,ab,kw. 6. esbl.ti,ab,kw. 7. methicillin-resistant staphylococcus aureus/ 8. methicillin-resistant staphylococcus aureus.ti,ab,kw. 9. methicillin resistance/ 10. methicillin resistance.ti,ab,kw. 11. mrsa.ti,ab,kw. 12. vancomycin-resistant enterococci/ 13. vancomycin-resistant enterococci.ti,ab,kw. 14. vancomycin resistance/ 15. vancomycin-resistant.ti,ab,kw. 16. vre.ti,ab,kw. 17. clostridium difficile/ 18. clostridium difficile.ti,ab,kw. 19. carbapenem-resistant enterobacteriaceae/ 20. carbapenem-resistant enterobacteriac*.ti,ab,kw. 21. cre.ti,ab,kw. 22. carbapenemase-producing enterobacteriac*.ti,ab,kw. 23. cpe.ti,ab,kw. 24. acinetobacter baumannii/ 25. acinetobacter baumannii.ti,ab,kw. 26. mdrab.ti,ab,kw. 27. mdr-ab.ti,ab,kw. 28. pseudomonas aeruginosa/ 29. pseudomonas aeruginosa.ti,ab,kw. 30. mdrpa.ti,ab,kw. 31. mdr-pa.ti,ab,kw. 32. klebsiella pneumoniae/ 33. klebsiella pneumoniae.ti,ab,kw.   **Long-term care facilities**   1. homes for the aged/ 2. nursing home/ 3. long term.ti,ab,kw. 4. Day Care, Medical/ 5. (elderly adj2 (home* or cent* or facilit* or unit* or institution*)).ti,ab,kw. 6. (geriatric adj2 (home* or cent* or facilit* or unit* or institution*)).ti,ab,kw. 7. (residential adj2 (home* or cent* or facilit* or unit* or institution*)).ti,ab,kw. 8. (retirement adj2 (home* or cent* or facilit* or unit* or institution*)).ti,ab,kw.   **Interventions**   1. infection control/ 2. infection control.ti,ab,kw. 3. standard care.ti,ab,kw. 4. personnel, hospital/ or hand disinfection/ or hand hygiene/ or hygiene/ or anti-infective agents, local/ 5. hand hygiene.ti,ab,kw. 6. contact precaution.ti,ab,kw. 7. antimicrobial stewardship/ 8. antimicrobial stewardship.ti,ab,kw. 9. disinfection/ 10. environment* clean*.ti,ab,kw. 11. decoloni#ation.ti,ab,kw. 12. decontamination/ 13. decontamination.ti,ab,kw. 14. equipment contamination/ 15. sanitation/ 16. patient isolation/ or social isolation/ 17. isolation.ti,ab,kw. 18. quarantine/ 19. quarantine.ti,ab,kw. 20. source control.ti,ab,kw. 21. mass screening/ 22. surveillance.ti,ab,kw. 23. screening.ti,ab,kw. 24. notification.ti,ab,kw. 25. cohorting.ti,ab,kw. 26. chlorhexidine.ti,ab,kw. 27. mandatory reporting.ti,ab,kw. 28. hand rub.ti,ab,kw. 29. glove*.ti,ab,kw.   **Outcomes of interest**   1. burden.ti,ab,kw. 2. mortality.ti,kw. 3. death*.ti,kw. 4. "costs and cost analysis"/ 5. cost*.ti,kw. 6. hospitali#ation.ti,kw. 7. length of stay.ti,kw. 8. productiv*.ti,kw. 9. morbidity.ti,kw. 10. coloni#ation.ti,ab,kw. 11. infection.ti,ab,kw. 12. acquisition.ti,ab,kw. 13. incidence/ 14. incidence.ti,ab,kw. 15. prevalence/ 16. prevalence.ti,ab,kw.   #87. OR (#1 to #33)  #88. OR (#34 to #41)  #89. OR (#42 to #70)  #90. OR (#71 to #86)  #91. AND (#87 to #90) | | |
| **CINAHL** | S78 | S27 AND S34 AND S52 AND S77 |  |
|  | S77 | S53 OR S54 OR S55 OR S56 OR S57 OR S58 OR S59 OR S60 OR S61 OR S62 OR S63 OR S64 OR S65 OR S66 OR S67 OR S68 OR S69 OR S70 OR S71 OR S72 OR S73 OR S74 OR S75 OR S76 |  |
|  | S76 | TI (incidence or prevalence) OR AB (incidence or prevalence) |  |
|  | S75 | (MM "Prevalence") |  |
|  | S74 | (MM "Incidence") |  |
|  | S73 | TI "acquisition" OR AB "acquisition" |  |
|  | S72 | MM "acquisition" |  |
|  | S71 | MM "acquisition" |  |
|  | S70 | TI "infection" OR AB "infection" |  |
|  | S69 | (MM "Infection") |  |
|  | S68 | TI "coloni#ation" OR AB "coloni#ation" |  |
|  | S67 | (MM "Bacterial Colonization") |  |
|  | S66 | TI "morbidity" OR AB "morbidity" |  |
|  | S65 | (MM "Morbidity") |  |
|  | S64 | TI "productiv*" OR AB "productiv*" |  |
|  | S63 | (MM "Productivity") |  |
|  | S62 | TI "length of stay" OR AB "length of stay" |  |
|  | S61 | (MM "Length of Stay") |  |
|  | S60 | TI "hospitali#ation" OR AB "hospitali#ation" |  |
|  | S59 | (MM "Hospitalization") |  |
|  | S58 | MM "cost*" |  |
|  | S57 | (MM "Costs and Cost Analysis") OR (MM "Health Care Costs") OR (MM "Nursing Costs") OR (MM "Economic Aspects of Illness") OR (MH "Cost Savings") OR (MM "Health Facility Costs") |  |
|  | S56 | TI (mortality or death*) OR AB (mortality or death*) |  |
|  | S55 | MM "mortality" |  |
|  | S54 | (MH "death*") |  |
|  | S53 | (MH "mortality") |  |
|  | S52 | S35 OR S36 OR S37 OR S38 OR S39 OR S40 OR S41 OR S42 OR S43 OR S44 OR S45 OR S46 OR S47 OR S48 OR S49 OR S50 OR S51 |  |
|  | S51 | TI "cohorting" OR AB "cohorting" |  |
|  | S50 | TI "surveillance" OR AB "surveillance" |  |
|  | S49 | (MH "Disease Surveillance") |  |
|  | S48 | MM "quarantine" |  |
|  | S47 | TI (isolation or quarantine) OR AB (isolation or quarantine) |  |
|  | S46 | MM "isolation" |  |
|  | S45 | TI "decontamination" OR AB "decontamination" |  |
|  | S44 | TI (sterili#ation or disinfection) OR AB (sterili#ation or disinfection) |  |
|  | S43 | (MH "Sterilization and Disinfection") |  |
|  | S42 | TI "Antimicrobial Stewardship" OR AB "Antimicrobial Stewardship" |  |
|  | S41 | (MM "Antimicrobial Stewardship") |  |
|  | S40 | TI (contact precaution* or universal precaution*) OR AB (contact precaution* or universal precaution*) |  |
|  | S39 | TI "hand washing" OR "hand washing" OR "hand hygiene" OR AB "hand washing" OR "hand washing" OR "hand hygiene" |  |
|  | S38 | (MM "hand washing" OR "hand washing" OR "hand hygiene") |  |
|  | S37 | TI "standard care" OR AB "standard care" |  |
|  | S36 | TI "infection control" OR AB "infection control" |  |
|  | S35 | (MM "infection control+") |  |
|  | S34 | S28 OR S29 OR S30 OR S31 OR S32 OR S33 |  |
|  | S33 | TI ("residential home*" OR "residential cent*" OR "residential facilit*" OR "residential institution*") OR AB "residential home*" OR "residential cent*" OR "residential facilit*" OR "residential institution*") |  |
|  | S32 | TI ("elderly home*" OR elderly cent*) OR AB ("elderly home*" OR elderly cent*) |  |
|  | S31 | TI "long term" OR AB "long term" |  |
|  | S30 | (MM "day care") |  |
|  | S29 | (MM "long term care") |  |
|  | S28 | (MM "health facilities+") |  |
|  | S27 | S1 OR S2 OR S3 OR S4 OR S5 OR S6 OR S7 OR S8 OR S9 OR S10 OR S11 OR S12 OR S13 OR S14 OR S15 OR S16 OR S17 OR S18 OR S19 OR S20 OR S21 OR S22 OR S23 OR S24 OR S25 OR S26 |  |
|  | S26 | TI "klebsiella pneumoniae" OR AB "klebsiella pneumoniae" |  |
|  | S25 | (MM "klebsiella") |  |
|  | S24 | TI mdr#pa OR AB mdr#pa |  |
|  | S23 | TI "pseudomonas aeruginosa" OR AB "pseudomonas aeruginosa" |  |
|  | S22 | (MM "pseudomonas infections") |  |
|  | S21 | TI mdr#ab OR AB mdr#ab |  |
|  | S20 | TI "acinetobacter baumannii" OR AB "acinetobacter baumannii" |  |
|  | S19 | (MM "acinetobacter infections") |  |
|  | S18 | TI "c* difficile" OR AB "c* difficile" |  |
|  | S17 | (MM "Clostridium Difficile") |  |
|  | S16 | TI vre OR AB vre |  |
|  | S15 | TI "vancomycin resistan*" OR AB "vancomycin resistan*" |  |
|  | S14 | TI mrsa OR AB mrsa |  |
|  | S13 | TI "methicillin resistan*" OR AB "methicillin resistan*" |  |
|  | S12 | TI esbl OR AB esbl |  |
|  | S11 | TI "extended spectrum beta#lactamase" OR AB "extended spectrum beta#lactamase" |  |
|  | S10 | ( TI cre OR AB cre ) OR ( TI cpe OR AB cpe ) |  |
|  | S9 | TI "carbapenem resistan*" |  |
|  | S8 | TI "carbapenem-producing enterobacteriaceae" OR AB "carbapenem-producing enterobacteriaceae" |  |
|  | S7 | TI "carbapenem-resistant enterobacteriaceae" OR AB "carbapenem-resistant enterobacteriaceae" |  |
|  | S6 | (MM "Methicillin Resistance") OR (MM "Methicillin-Resistant Staphylococcus Aureus") OR (MM "Vancomycin Resistant Enterococci") OR (MM "Carbapenem-Resistant Enterobacteriaceae") |  |
|  | S5 | TI amr OR AB amr |  |
|  | S4 | TI mdro OR AB mdro |  |
|  | S3 | TI "antimicrobial resistan*" OR AB "antimicrobial resistan*" |  |
|  | S2 | TI "multi#drug resistan*" OR AB "multi#drug resistan*" |  |
|  | S1 | (MM "drug resistance+") |  |

|  |
| --- |
