# Supplementary material for: Approaches to multidrug-resistant organism prevention and control in long-term care facilities for older people: a systematic review and meta-analysis
Source: Antimicrob Resist Infect Control. 2022 Jan 15;11:7. doi: 10.1186/s13756-021-01044-0 (PMC8761316; doi:10.1186/s13756-021-01044-0)
Supplement: Supplementary file 2 — Additional file 2. Description of infection prevention and control strategies. [file 13756_2021_1044_MOESM2_ESM.docx]

**Additional file 2. Description of infection prevention and control strategies.**

| **Intervention** | **Details** |
| --- | --- |
| Administrative engagement (AE) | Administrative engagement refers to the active involvement of the administration in planning, implementing, and providing feedback on infection prevention control strategies. |
| Barrier precautions (BP) | Barrier precautions are a collection of horizontal infection control interventions to prevent transmission of infectious agents spread by direct or indirect contact with the patient or potentially contaminated environment. Examples include wearing personal protective gears (i.e., don gowns, caps, and gloves); patient isolation or cohorting (i.e., confine patients colonized or infected with the same infectious agent in the same area); and staff cohorting (i.e., assign dedicated staff to colonized or infected patients). . |
| Decolonization (DC) | Decolonization is a vertical infection control intervention to eliminate a specific type of infectious agent. It applies to the colonized residents with a specific regimen consisting of antimicrobial agents, which can be administered in various forms: ointment, oral paste, and digestive solution. |
| Education (ED) | Education refers to health training targets to healthcare staff on improving infection control practices (e.g., posters, leaflets, training sessions, practical demonstrations on hand hygiene and decontamination of equipment and the environment). |
| Environmental cleaning (EC) | Environmental cleaning applies to cleaning and disinfection of all medical devices and patient-care areas (e.g., bed rails, bedside tables, commodes, doorknobs, sinks, surfaces, and equipment close to the patient). |
| Hand hygiene (HH) | Hand hygiene applies to any one of the following practices: 1) handwashing with plain (non-antimicrobial) soap and water; 2) antiseptic hand wash (soap containing antiseptic agents and water); 3) antiseptic hand rub; 4) revision of hand hygiene technique with demonstration; 5) reminders of hand hygiene at opportunities, 6) reinforcing hand hygiene, or 7) the authors stated hand hygiene was one of the components in the interventions. Our review did not classify hand hygiene education alone as a hand hygiene intervention. |
| Performance improvement (PI) | Performance improvement applies to any form of audits on the performance of current infection control strategies with written and verbal feedback from an infection control expert (e.g., infection control nurse), which detailed infection control practice and how the current practice can be improved. |
| Source control (SC) | Source control applies to daily bathing, showering, or whole-body washing with chlorhexidine gluconate. |
| Usual care (UC) | Usual care refers to the standard practices from the facility. |
