# Supplementary material for: Approaches to multidrug-resistant organism prevention and control in long-term care facilities for older people: a systematic review and meta-analysis
Source: Antimicrob Resist Infect Control. 2022 Jan 15;11:7. doi: 10.1186/s13756-021-01044-0 (PMC8761316; doi:10.1186/s13756-021-01044-0)
Supplement: Supplementary file 3 — Additional file 3. Interventions included in each studies. [file 13756_2021_1044_MOESM3_ESM.docx]

**Appendix 3. Interventions included in each studies.**

| **Reference** |  | **AE** | **BP** | **DC** | **EC** | **ED** | **HH** | **PI** | **SC** | **UC** |
| --- | --- | --- | --- | --- | --- | --- | --- | --- | --- | --- |
| Baldwin et al. (2010) |  | O | 🞨 | 🞨 | 🞨 | 🗸 | 🞨 | 🗸 | 🞨 | 🗸 |
| Bellini et al. (2015) |  | 🞨 | 🞨 | 🗸 | 🗸 | 🗸 | 🞨 | 🞨 | 🞨 | 🗸 |
| Ben-David et al. (2019) |  | 🗸 | 🗸 | 🞨 | 🞨 | 🗸 | 🞨 | 🗸 | 🞨 | 🗸 |
| Bowler et al. (2010) |  | 🞨 | 🞨 | 🗸 | 🗸 | 🗸 | 🞨 | 🞨 | 🞨 | 🗸 |
| Chuang et al. (2015) |  | O | 🗸 | 🞨 | 🗸 | 🗸 | 🗸 | 🗸 | 🞨 | 🗸 |
| Hequet et al. (2017) |  | 🞨 | 🞨 | 🗸 | 🗸 | 🗸 | 🞨 | 🞨 | 🞨 | 🗸 |
| Ho et al. (2012) |  | 🗸 | 🞨 | 🞨 | 🞨 | 🗸 | 🗸 | 🗸 | 🞨 | 🗸 |
| Horner et al. (2012) |  | 🞨 | 🞨 | 🞨 | 🞨 | 🗸 | 🞨 | 🗸 | 🞨 | 🗸 |
| Jaqua-Stewart et al. (1999) |  | 🞨 | 🗸 | 🗸 | 🞨 | 🗸 | 🞨 | 🞨 | 🗸 | 🗸 |
| Kauffman et al. (1993) |  | 🞨 | 🞨 | 🗸 | 🞨 | 🞨 | 🞨 | 🞨 | 🞨 | 🗸 |
| Mody et al. (2015) |  | 🞨 | 🗸 | 🞨 | 🞨 | 🗸 | 🗸 | 🗸 | 🞨 | 🗸 |
| Morgan et al. (2019) |  | 🞨 | 🗸 | 🞨 | 🞨 | 🞨 | 🞨 | 🞨 | 🞨 | 🗸 |
| Ostrowsky et al. (2001) |  | 🞨 | 🗸 | 🞨 | 🗸 | 🗸 | 🗸 | 🞨 | 🞨 | 🗸 |
| Peterson et al. (2016) |  | 🞨 | 🞨 | 🗸 | 🗸 | 🗸 | 🞨 | 🞨 | 🗸 | 🗸 |
| Schora et al. (2014) |  | 🞨 | 🞨 | 🗸 | 🗸 | 🗸 | 🞨 | 🞨 | 🗸 | 🗸 |
| Schweon et al. (2013) |  | 🗸 | 🞨 | 🞨 | 🞨 | 🗸 | 🗸 | 🗸 | 🞨 | 🗸 |
| Silverblatt et al. (2000) |  | 🞨 | 🗸 | 🗸 | 🞨 | 🗸 | 🗸 | 🞨 | 🞨 | 🗸 |
| Singh et al. (2018) |  | 🗸 | 🗸 | 🞨 | 🗸 | 🗸 | 🗸 | 🞨 | 🞨 | 🗸 |
| Thomas et al. (1989) |  | 🞨 | 🗸 | 🞨 | 🞨 | 🗸 | 🞨 | 🞨 | 🞨 | 🗸 |

AE, administrative engagement; BP, barrier precautions; DC, decolonization; ED, education; EC, environmental cleaning; HH, hand hygiene; PI, performance improvement; SC, source control; UC, usual care

🗸 indicates the intervention was included in the study

🞨 indicates the intervention was not included in the study

O indicates the authors emphasized that the intervention was not provide
