# Supplementary material for: Approaches to multidrug-resistant organism prevention and control in long-term care facilities for older people: a systematic review and meta-analysis
Source: Antimicrob Resist Infect Control. 2022 Jan 15;11:7. doi: 10.1186/s13756-021-01044-0 (PMC8761316; doi:10.1186/s13756-021-01044-0)
Supplement: Supplementary file 4 — Additional file 4. Characteristics of included studies. [file 13756_2021_1044_MOESM4_ESM.docx]

| Additional file 4. Characteristics of included studies. | | | | | | | | |
| --- | --- | --- | --- | --- | --- | --- | --- | --- |
| Reference, country | **Study year, duration** | **Study design** | **Setting, no. of facilities analyzed^^[[1]](#footnote-1)^^** | **Interventions evaluated** | **Compliance** | **No. of residents analyzed** | **Measured outcomes reported** | **Conclusions** |
| Baldwin et al. (2010), Northern Ireland | 2007, 20mths | Clustered RCT, matched  Concurrent control | 32 NHs  n_Int._=16;  n_cnt._=16 | **(1) Education (2) Performance Improvement**  Int.:  - Perform active surveillance  - Provide an IPC training  - Demonstrate HH and decontamination of equipment and environment to staff  - Provide an audit score assessing the compliance  - Provide feedback to the manager  - Appoint selected HCWs  with additional training to reinforce all aspects of good IPC  - Repeat IPC training and demonstration at three and six months after each audit  Cnt.:  - Perform active surveillance  - Continue the usual care | **HH**  **Baseline**  =30.5%  **Post-int. (3mths)**  Int.=74%  Cnt.=57%  **Post-int. (6mths)**  Int.=81%  Cnt.=63%  **Post-int. (12mths)**  Int.=82%  Cnt.=64% | N=793  n_Int._=392;  n_cnt._=401 | **MRSA (N,U,W) colonization**  **Prevalence**  **Pre-int.**  Int.=67/392 (17.1%)  Cnt.=68/401 (16.7%)  **Post-int. (3mths)**  Int.=77/312 (24.7%)  Cnt.=81/315 (25.7%)  *RR=0.98 (0.74,1.29) ;p=0.87*  **Post-int. (6mths)**  Int.=55/272 (20.2%)  Cnt.=67/271 (24.7%)  *RR: 0.83 (0.60,1.16); p=0.28*  **Post-int. (12mths)**  Int.=44/234 (18.8%)  Cnt.=47/244 (19.3%)  *RR: 0.99 (0.69,1.42); p=0.95* | IPC program did not affect MRSA prevalence among residents over the 12-month study period despite significantly higher infection control audit scores from the intervention group.  Poor administrative engagement is reported. Despite regular feedback from the infection control nurse to the managers on the poor audit results, there were limited attempts by management to address non-compliance. |
| Bellini et al. (2015)[]](https://paperpile.com/c/ivsOrE/YP8Z), Switzerland | 2010, 19mths | Clustered RCT  Concurrent control | 104 NHs  n_Int._=53;  n_cnt._=51 | **(1) Decolonization (2) Education (3) Environmental cleaning**  Int.:  - Perform active surveillance  - Provide training on standard precaution to all HCWs  - Decolonize MRSA positive residents for over five consecutive days (nostrils, pharynx, hair, skin)  - Disinfect the environment  - Provide specific training and teaching material about the decolonization protocol and environmental disinfection to HCWs  Cnt.:  - Perform active surveillance  - Provide training on standard precaution to all healthcare workers | NS | N=4750  n_Int._=2338;  n_cnt._=2412 | **MRSA (N,I,W,U) colonization**  **Prevalence**  **Pre-int.**  Int.=8.9%  Cnt.=8.9%  **Post-int.(12mths)**  Int.=5.8%  Cnt.=6.6%  **Infection**  No invasive MRSA infections were observed during the entire study period. | IPC program had no effect on MRSA prevalence among residents. MRSA prevalence declined significantly over the study period in both the intervention and control groups. |
| Ben-David et al. (2019), Israel | 2009, 84mths | Longitudinal, uncontrolled before-after study  Historical control | 300 NHs | **(1) Administrative engagement (2) Barrier precaution (3) Education (4) Performance improvement**  - Perform active surveillance  - Initiate and coordinate by the Ministry of Health  - Administrative involvement in continuous supervision  - Provide IPC training^^[[2]](#footnote-2)^^  - Implement contact precaution  - Send periodic reports to the CDC  - Reinforce early case detection of cases and prompt case reporting  - Supervise information exchange between healthcare facilities | NS | N=20000^^[[3]](#footnote-3)^^ | **CRE (R) acquisition**  No. of acquisition = 2182  **Incidence of acquisition (/10 000 patient-days):**  Pre-int.=0.5  Post-int.(84mths)=0.3  **IRR (95% CI)**  = 0.93 (0.91-0.95), p<0.001 | The national, coordinated intervention resulted in a sustained decrease in CRE incidence and prevalence in LTCFs. |
| Bowler et al. (2010), US | 2006, 13mths | Uncontrolled before-after study  Historical control | 5 NHs | **(1) Decolonization (2) Education (3) Environmental cleaning**  - Perform active surveillance  - Decolonize MRSA positive residents  - Decontaminate the rooms of MRSA positive residents  - Educate patients | NS | N=687  n_pre-int.._=687;  n_post-int._=653 | **MRSA (N,W,U) colonization**  **Prevalence**  Pre-int.=80/687 (11.6%)  Post-int.(13mths)=26/653 (4.0%)  *RR=0.48 (0.34,0.68);p<0.001* | The use of active surveillance cultures and decolonization therapy effectively decreased the prevalence of MRSA in nursing homes. |
| Chuang et al. (2015), Hong Kong SAR | 2010, 16mths | Clustered RCT  Concurrent control | 36 NHs  n_Int._=18;  n_cnt._=18 | **(1) Barrier precaution (2) Education (3) Environmental cleaning (4) Hand Hygiene (5) Performance improvement**  Int.:  - Introduce and install alcohol-based hand rubs at designated areas^^[[4]](#footnote-4)^^  - Display poster demonstrating the Five Moments of correct hand hygiene indications and the Seven Steps of effective hand hygiene techniques from the World Health Organization  - Provide color-coded cleaning kits along with protocol to the NHs  - Isolate MRSA carriers from vulnerable residents who had indwelling catheters or skin lesion  - Reinforce contact precaution when handling the device and wounds of MRSA carrier  - Allow MRSA carriers without any wounds or indwelling catheters to participate in social activities  - Assess staff compliance with the interventions quarterly together with timely feedback to the stakeholders  Cnt.:  - Continue the usual practice | **HH**  **Pre-int.**  Int.=5.9%  Cnt.=5.9%  **Post-int. (15mths)**  Int.=45.6%  Cnt.=7.2% | N=2776  n_Int._=1505;  n_cnt._=1271 | **MRSA (Unk) colonization**  **Prevalence**  **Pre-int.**  Int.=316/1505 (21.0%)  Cnt.=249/1271 (19.6%)  *OR: 1.11 (0.86,1.44); p=0.42*  **Post-int. (6mths)**  Int.=341/1480 (23.0%)  Cnt.=239/1226 (19.5%)  *OR: 1.18 (0.87,1.60); p=0.30*  **Post-int.(9mths)**  Int.=304/1469 (20.7%)  Cnt.=231/1184 (19.5%)  *OR: 1.06 (0.81,1.38); p=0.69*  **Post-int. (12mths)**  Int.=303/1446 (21.0%)  Cnt.=243/1159 (21.0%)  *OR: 0.99 (0.74, 1.34); p=0.95*  **Post-int. (15mths)**  Int.=290/1367 (21.2%)  Cnt.=229/1116 (20.5%)  *OR: 1.04 (0.72, 1.49); p=0.85* | The study showed that relying on the infection control bundle alone could not bring sustainable MRSA reduction.  Administrative control for strengthening infection control infrastructure is important for continuous compliance and improvement. |
| Hequet et al. (2017), Switzerland  **Extended study from Bellini et al. (2015)* [*[2]*](https://paperpile.com/c/ivsOrE/YP8Z) | 2010, 72mths | Clustered RCT  Concurrent control | 92 NHs  n_Int._=47;  n_cnt._=45 | **(1) Decolonization (2) Education (3) Environmental cleaning**  Int.:  - Perform active surveillance  - Provide training on standard precaution to all HCWs  - Decolonize MRSA positive residents for over five consecutive days  - Disinfect the environment  Cnt.:  - Active surveillance  - Provide training on standard precaution to all HCWs | NS | NS | **MRSA (N,P,I) colonization**  **Prevalence^^[[5]](#footnote-5)^^**  **Pre-int.**  Int.=8.3%  Cnt.=9.0%  *(p=0.60)*  **Post-int. (12mths)**  Int.=5.4%  Cnt.=6.3%  *(p=0.18)*  **Post-int.(60mths)**  Int.=4.0%  Cnt.=8.1%  *(p=0.01)* | A five-year follow-up shows no evidence supporting IPC interventions. |
| Ho et al. (2012), Hong Kong SAR | 2007, 43mths | Clustered RCT  Concurrent control | 18 EHs  n_Int1._=6;  n_Int2._=6;  n_cnt._=6 | **(1) Administrative engagement (2) Education (3) Hand hygiene (4) Performance improvement**  Int.1:  - Supply slightly powered gloves  - WHO multimodal strategy^^[[6]](#footnote-6)^^  Int.2:  - Supply powderless gloves  - WHO multimodal strategy*  Cnt.:  - Deliver a two-hour health talk on personal, food, and environmental hygiene, healthy eating, and regular exercise. HH was mentioned as a component of personal hygiene, but nothing was said about HH indications | **HH^^[[7]](#footnote-7)^^**  **Pre-int.**  Int.1=325/1204 (27.0%)  Int.2=313/1410 (22.2%)  Cnt.=326/1671 (19.5%)  **Post-int. (1mths)**  Int.1=699/1181 (59.2%)  Int.2=763/1274 (59.9%)  Cnt.=299/1508 (19.8%)  **Post-int. (4mths)**  Int.1=662/1093 (60.6%)  Int.2=454/935 (48.6%)  Cnt.=301/1393 (21.6%) | N=2407  n_Int1._=767; n_Int2._=929;  n_cnt._=711 | **MRSA infections requiring hospitalization**  **Pre-int.**  Int.1=21/767 (2.7%)  Int.2=32/929 (3.4%)  Cnt.=24/711 (3.4%)  **Post-int. (4mths)**  Int.1=15/767 (2.0%)  Int.2=47/929 (5.1%)  Cnt.=31/711 (4.4%)  **IRR of MRSA infections requiring hospital admission**  *IRR_MRSA hospital admission_ = 0.61 (0.38, 0.97); p=0.04* | The WHO multimodal strategy effectively reduces MRSA infections requiring hospitalization in the homes for the elderly. |
| Horner et al. (2012), UK | 2006, 28 months | Controlled before-after study (stepped wedge design)  Historical control | 68 EHs  n_Int.1_=28;  n_Int.2_=18;  n_cnt._=22 | **(1) Education (2) Performance improvement**  - Perform active surveillance  - Provide structured session of education on infection prevention and effective hand hygiene  - Audit hand hygiene practice, staff education, compliance with requirements, and facilities in EHs  - Give written feedback to each EH | **HH^^[[8]](#footnote-8)^^**  **Pre-int.**  = 58% of 568 opportunities  **Post-int.**  = 82% of 455 opportunities | N=2227^^[[9]](#footnote-9)^^ | **MRSA (N) colonization**  **Prevalence^^[[10]](#footnote-10)^^**  **Pre-int.**  Group 1=0.20 (20%)  Group 2=0.19 (19%)  Group 3=0.19 (19%)  **Post-int. (6mths)**  Group 2=0.28 (28%)  Group 3= 0.24 (24%)  **Post-int. (9mths)**  Group 1=0.24 (24%)  **Post-int. (12mths)**  Group 2=0.19 (19%)  **Post-int. (18mths)**  Group 1=0.22 (22%)  Group 2=0.18 (18%)  **Post-int. (24mths)**  Group 1=0.22 (22%) | The intervention was associated with a small but significant increase in MRSA prevalence. |
| Jaqua-Stewart et al. (1999), US | 1993, 39mths | Uncontrolled before-after study  Historical control | A NH | (**1) Barrier precaution (2) Decolonization (3) Education (4) Source control**  - Performed active surveillance  - Apply contact isolation for MRSA positive residents  - Bath with Chlorhexagluconate for MRSA positive residents  - Decolonize nasal carriers with mupirocin  - Treat with two oral antibiotics for MRSA colonized residents (other than nasal)  - Educate all interdisciplinary staff regarding MRSA, hand washing, program, objectives, etc. | NS | N=42 | **MRSA (N,Sp,U) colonization**  **MRSA (N,Sp,U) infections**  **Prevalence of colonization**  Pre-int.=22/42 (52.4%)  Post-int.(12mths)=2%  **Prevalence of infections**  Pre-int.=8.5% (1993 year)  Pre-int.=33% (Oct 1993)  Post-int.(39mths)=1.4% | Aggressive containment practices applied to a nursing home with a high MRSA infection rate not only reduced rates of colonization but also markedly reduced infections. This reduction was maintained over time. The cost of the containment practice was cost-effective. |
| Kauffman et al. (1993), US | 1990, 12mths | Uncontrolled before-after study  Historical control | 1 NH | **(1) Decolonization**  Phase 1: Only treated patients colonized with MRSA in their anterior nares (7mths)  - Perform active surveillance  - Apply daily mupirocin 2% ointment on nares for decolonization  - Apply ointment on nares daily for one week; 3 times a week for 3 weeks; once a week for 3 months till all cultures are negative  Phase 2: Treat patients colonized with MRSA in their anterior nares and wounds (7mths)  - Perform active surveillance  - Apply daily mupirocin 2% ointment on both nares and wounds for decolonization for MRSA colonized residents  - Apply ointment on nares and wounds daily for two weeks; three times a week for three weeks; once a week for three months till all cultures are negative | NS | N=321 | **MRSA (N,W) colonization**  **Prevalence**  Phase 1  Pre-int.=15.2%  Post-int.(7mths)=12.0%  Phase 2  Pre-int.=15.2%  Post-int.(5mths)=6.3%  **MRSA infections^^[[11]](#footnote-11)^^**  Pre-int.= 9/341 (2.6%)^^[[12]](#footnote-12)^^  Post-int.(11mths)=6/321 (1.9%)  Phase 1  Post-int.(7mths)= 3/321 (0.9%)  Phase 2  Post-int.(5mths)= 3/321 (0.9%) | Mupirocin ointment applied on both nares and wounds is effective at decreasing colonization with MRSA in nursing homes. However, it should be saved for use in outbreak situation, and not used over the long term in facilities with endemic MRSA colonization |
| Mody et al. (2015), US | 2010, 36mths | Clustered RCT  Concurrent control | 12 NHs  n_Int._=6;  n_cnt._=6 | (**1) Barrier precaution (2) Education (3) Hand hygiene (4) Performance improvement**  Int.:  - Place on preemptive barrier precautions for individual with an indwelling device (glove and gown use for direct care)  - Place barrier precaution sign on the doors to their rooms, inside their closet, at the nurses’ station, and on their medical records  - Promote hand hygiene and barrier precaution among HCWs using posters, video, Glo Germ gel, pre-post culture, interactive infection prevention modules, infection surveillance pocket cards  - Encourage HCWs to perform hand hygiene before and after providing any care to the participants  - Place hand hygiene products and personal protective equipment strategically at each site  - Not isolate any residents (allow socialization and obtain rehabilitation outside their room)  - Active surveillance with data reported back to the facilities every month, along with reminders of key strategies to prevent infections  Cnt.:  - Standard precaution  - Passive surveillance^^[[13]](#footnote-13)^^  - Education provided as needed | A structured 30-minute observation to monitor HCW activities and their use of barrier precautions but results were not reported. | N=418 (34174 device-days);  n_Int._=203; 17490 device-days; 3283 active surveillance samples  n_Cnt.=_215; 16684 device-days; 3274 active surveillance sample | **All MDRO (MRSA, VRE, CFT-R GNB, CIP-R GNB) (C,F,I,N,P,R,W) colonization**  **Prevalence^^[[14]](#footnote-14)^^**  All MDRO  Int.=1299/4883 (26.6%)  Cnt.=1732/5313 (32.6%)  MRSA  Int.=254/3098 (8.2%)  Cnt.=323/3076 (10.5%)  VRE  Int.= 122/3128 (3.9%)  Cnt.=162/3057 (5.3%)  CTZ-R GNB  Int.=185/3426 (5.4%)  Cnt.=295/3512 (8.4%)  CIP-R GNB  Int.=738/3785 (19.5%)  Cnt.=952/3934 (24.2%)  **Prevalence density rate^^[[15]](#footnote-15)^^:**  All MDRO = 0.77 (0.62-0.94)  MRSA = 0.78 (0.64-0.96)  VRE = 1.20 (0.82-1.75)  CTZ-R GNB = 0.94 (0.61-1.44)  CIP-R GNB = 0.75 (0.58-0.97)  **Risk of new MDRO acquisition (/1000 device-days at risk)^^[[16]](#footnote-16)^^**  MRSA  Int.=54/8772 (0.62%)  Cnt.=56/7115 (0.79%)  VRE  Int.=22/12756 (0.17%)  Cnt.=26/11070 (0.23%)  CTZ-R/CIP-R GNB  Int.=42/7524 (0.56%)  Cnt.=35/5685 (0.62%)  **Cluster- and covariate-adjusted Hazard Ratio for new acquisition (95%CI)**  MRSA = 0.78 (0.65-0.95)  VRE = 0.85 (0.45-1.60)  CIP-R/ CTZ-R GNB = 0.90 (0.60-1.33)  **Incidence rates of device-associated infections (/1000 device-days at risk)**  New CAUTI  Int.=31/5982 (0.52%)  Cnt.=43/4292 (1.00%)  New & recurrent CAUTI  Int.=56/9413 (0.59%)  Cnt.=75/8118 (0.92%)  Feeding tube associated SSTI Int.= 4/5635 (0.07%)  Cnt.=3/5062 (0.06%)  Feeding tube-associated pneumonia  Int.=10/5635 (0.18%)  Cnt.=8/5062 (0.16%)  **Cluster- and covariate-adjusted hazard ratio (95%CI)**  New CAUTI=0.54 (0.30-0.97)  New & recurrent CAUTI = 0.69 (0.49-0.99)  Feeding tube associated SSTI =1.09 (0.22-5.45)  Feeding tube-associated pneumonia =1.83 (0.53-6.31) | The intervention bundle reduced the overall MDRO prevalence density, new MRSA acquisitions, and clinically defined catheter-associated urinary tract infection rates in high-risk NH residents with indwelling devices. |
| Morgan, et al. (2019), US | 2011, 48 months | Controlled before-after study  Concurrent control | 74 LTCFs  n_Int._=45;  n_cnt._=28 | **(1) Barrier precaution**  Int.:  - Apply contact precaution  - Use of gowns and gloves for all contact with residents or with the environment within their rooms for those residents colonized or infected with MRSA  Cnt.:  - Apply standard precaution (basic self-hygiene) | NS | N=75414  n_Int._=45167;  n_Cnt.=_30247 | **MRSA (Unk) acquisition**  **MRSA associated infections**  **Prevalence (/1000 patient-days)**  Int.= 2.55 (0.25%)  Cnt.= 2.54 (0.26%)  **Infections (/1000 patient-days)^^[[17]](#footnote-17)^^**  Int.= 0.14 (0.01%)  Cnt.= 0.11 (0.01%) | Contact precaution was not more effective in reducing MRSA acquisition and infection compared to standard precaution. |
| Ostrowsky et al. (2001), US | 1997, 28 months | Uncontrolled before-after study  Historical control | 28 LTCFs | (**1) Barrier precaution (2) Education (3) Environmental cleaning (4) Hand hygiene**  - Perform active surveillance  - Screen actively on admission to identify VRE positive residents  - Cohort/ isolate for VRE positive residents  - Apply contact precaution with gloves and gown for direct contact  - Encourage hand washing to health care workers, residents, and visitors  - Dedicate use of noncritical equipment  - Clean and disinfect equipment with approved disinfectants  - Provide education materials to health care workers, patients, and visitors  - Facilitate communication within facilities by stating the VRE status of patients orally or transfer sheet | Y^^[[18]](#footnote-18)^^  Compliance for isolation/ cohorting interventions (No. of LTCFs)  Year 1 = 21/23 (91%)  Year 2 = 22/25 (88%) | N=5221  Pre-int.= 1782  Post-int.=1789  Post-int.= 1650 | **VRE (R) colonization**  **Prevalence**  Pre-int.= 30/1782 (1.7%)  Post-int.(12mths) = 17/1789 (1.0%)  Post-int.(24mths) = 9/1650 (0.5%)  **Relative risk**  Pre-int. vs. Post-int.(12mths)  = 0.6 (0.3-1.0); p=0.05  Pre-int. vs. Post-int. (24mths)  0.3 (0.2-0.7); p=0.001 | An active infection-control intervention, which includes the obtaining of surveillance cultures and the isolation of infected patients, can reduce or eliminate the transmission of vancomycin- resistant enterococci in LTCFs. |
| Peterson et al. (2016), US  *Schora et al. (2014) and Peterson et al. (2016) are based on the same study with the same study sample.* | 2011, 25mths | Prospective, cluster RCT, non-blinded  Concurrent control | 3 LTCFs | **(1) Decolonization (2) Education (3) Environmental cleaning (4) Source control**  **Year 1**  Int.:  - Perform active surveillance  - Test all new admission on-site followed by decolonization of those positive  - Decolonize all residents with mupirocin ointment twice at the onset of the study and once at the beginning and again at the end of March 2011  - Provide education on the nature of pathogen transmission, the need for effective cleaning and disinfection of healthcare facility surfaces/ equipment, and the importance of hand hygiene  - Patients who had MRSA strains resistant to mupirocin were given retapamulin 1% in the same manner as mupirocin  - Use at least one chlorhexidine gluconate 4% body wash in the five-day regime  - Decontaminate the environmental flat surfaces in all rooms, common areas, and equipment with bleach for over a one-week period before each point prevalence survey  Cnt.:  - Perform active surveillance  **Year 2**  Int. & Cnt.:  - Perform active surveillance  - Convert all units to intervention consisting of universal decolonization^^[[19]](#footnote-19)^^  - Retest all MRSA positive residents after decolonization and this process was continued until they had a negative test for MRSA in their nares or were discharged  - Use intensive decolonization regimen for those not decolonized with nasal treatment alone (the regimen consisted of minocycline 100mg orally twice daily for 5 days; rifampin 600mg orally once daily for 5 days; 2% mupirocin ointment to the anterior nares twice per day for 7 days, and a bath or shower with 4% chlorhexidine once per week for 2 weeks)  - See Year 1 | NS | N=7069 | **MRSA infections**  **Infection rates (/10,000 patient-days)^^[[20]](#footnote-20)^^**  Pre-int.:  Int.&Cnt.=44/365809 =1.20  Post-int.(12mths):  Int.=14/129113 =1.08  Cnt.=9/165052 = 0.55  Post-int.(24mths)  Int.&Cnt.=12/287847=0.42  (p<.001 compared to baseline) | On-site MRSA surveillance with targeted decolonization resulted in a significant decrease in clinical MRSA infection among LTCF residents. |
| Schora et al. (2014), US^^[[21]](#footnote-21)^^ | 2011, 25mths | Prospective, cluster RCT  Concurrent control | 3 LTCFs^^[[22]](#footnote-22)^^ | **(1) Decolonization (2) Education (3) Environmental cleaning (4) Source control**  **Year 1^^[[23]](#footnote-23)^^**  Int.:  - Perform active surveillance  - Test new admission on-site and decolonized those tested positive  - Decolonize all residents with mupirocin ointment twice a day for five days (applied to the nares)  - Apply chlorhexidine bath once per week (repeat the regime a second time 1 month later)  - Clean the common areas, nursing units, equipment, and patient rooms with bleach  - Provide hand hygiene education to all health care personnel  - Test all discharge for nasal MRSA colonization in the last four month of year 1  - Apply Chlorhexidine bathing  Cnt.:  - Perform active surveillance  - Test all new admission on-site without reporting the results to the staff and taking any further action  **Year 2**  Int. & Cnt.:  - All units were converted to intervention group^^[[24]](#footnote-24)^^  - See Year 1^^[[25]](#footnote-25)^^ | NS | N=4424 | **MRSA (N) colonization**  **Prevalence**  Pre-int.:  Int.=53/315 (16.8%)  Cnt.=59/358 (16.5%)  Post-int. (12mths)  Int.=83/715 (11.6%) *(p=0.028)*  Cnt.=136/762 (17.9%) *(p=0.613)*  Post-int. (24mths)^^[[26]](#footnote-26)^^  Int. = 240/2274 (10.6%) *(p<.001)* | The planned interventions of screening and decolonization were successful at lowering MRSA colonization without affecting the home-like style of living of LTCF residents. |
| Schweon et al. (2013), US | 2009, 22 months | Uncontrolled interrupted time series study  Historical control | A NH | **(1) Administrative engagement (2) Education (3) Hand hygiene (4) Performance improvement**  - Place touch-free dispensers containing hand sanitizer foam in high-traffic areas throughout the facility  - Implement an education program for all HCP  - Present CDC HH guidelines on the timing of performing hand hygiene  - Present videos demonstrating proper hand hygiene techniques  - Distribute instant hand sanitizer for personal carriage to all HCPs upon completion of the education program  - Mount posters promoting hand hygiene at strategic location to serve as reminders  - Identify and reward the HCP with the best compliance  - Create a poster containing the champion’s quote about the importance of hand hygiene, a picture of him/her using the ABHR  - Involve the infection preventionist and the Director of Nursing in promoting hand hygiene in all resident care areas for all shifts  - Provide educational information about hand hygiene program to residents at the monthly Resident’s Council meeting  - Promote hand hygiene in all resident care areas for all shifts from the infection preventionist and the Director of Nursing | Monthly HH compliance monitoring^^[[27]](#footnote-27)^^  Staff compliance^^[[28]](#footnote-28)^^ = 259/480=54%  *480 direct observation | NS | **MRSA incident infection rates**  **MRSA incident infection rates (/1000 resident rate)**  Pre-int.=0.53  Post-int.=0.55  *(p=0.89)*  **VRE incident infection rate C. difficile incident infection rates**  **VRE incident infection rates (/1000 resident rate)**  Pre-int.=0.07  Post-int.=0.05  *(p=0.80)*  **C. difficile incident infection rates**  **C. difficile incident infection rates (/1000 resident days)**  Pre-int.=0.08  Post-int.=0.04  *(p=0.36)* | The comprehensive hand hygiene program was not effective in reducing MRSA-, VRE-, and C.diff-related infections in the LTCF. |
| Silverblatt et al. (2000), US | 1996, 32mths | Uncontrolled interrupted time series study  Historical control | A NH | **(1) Barrier precaution (2) Decolonization (3) Education (4) Hand hygiene**  - Perform active surveillance  - Screen for VRE on admission and discharge  - Isolate colonized/infected residents  - Use oral bacitracin to decolonize VRE positive residents  - Repeat rectal swab for VRE-positive residents monthly until negative  - Place VRE-colonized or -infected residents in individual rooms or cohort with another VRE-positive resident  - Allow residents to leave their rooms if they were competent to follow hand washing instructions  - Allow residents to leave their rooms with supervision if they were not competent to follow hand washing instruction  - Wear clean clothes and leave all personal articles inside their rooms, including wheelchairs, outside their rooms  - Instruct caregiver to don gowns and gloves before entering the room of colonized residents  - Enforce hand washing strictly for personnel  - Require personnel to rinse with an alcohol-based waterless skin disinfectant after washing  - Give all staff and family members instructions on the isolation techniques  - Label rooms suitably at their entrance | NS | N=199 | **VRE (R) colonization**  **Prevalence^^[[29]](#footnote-29)^^**  Pre-int.=0/69  Post-int.=0/130 | Adherence to infection control practices by the patient care staff of the LTCF was associated with the absence of transmission of VRE colonization among its residents. The presence of rectal colonization with VRE in an acute care patient should not be a barrier to acceptance in a nursing home. |
| Singh et al. (2018), US | 2014, 33 mths | Uncontrolled before-after study  Historical control | 132 Veterans Affairs facilities | **(1) Administrative engagement (2) Barrier precaution (3) Environmental cleansing (4) Education (5) Hand hygiene**  - Clean the environmental  - Perform hand hygiene  - Perform contact precautions for suspected or documented C.diff. cases  - An institutional culture change in which infection control becomes everyone’s business | NS | N=9288098 resident days | **C.diff. infection^^[[30]](#footnote-30)^^ (/10000 resident days)**  Pre-int.=1.98  Post-int.(33mths)=1.26 | The nationwide number of clinically confirmed LTCF-onset C.diff. cases decreased 36.1% in the subsequent 33-month analysis period. |
| Thomas et al. (1989), US | 1987, 3mths | Uncontrolled before-after study  Historical control | A LTCF | **(1) Barrier precaution (2) Education**  - Move MRSA positive residents into a designated ward  - Assign employees MRSA cases into the same ward  - Educate the employees on hygiene measures to prevent transmission of the organism | NS | N=164 | **MRSA (N) colonization**  **Prevalence**  Pre-int.=12/164 (7.3%)  Post-int.(3mths)=10/166 (6.0%)  **MRSA-related infections**  Pre-int.=3/164 (1.8%)  Post-int.=1/166 (0.6%) | Modest control measures reduced MRSA colonization and its related infections among residents in the LTCF. |
|  |  |  |  |  |  |  |  |  |
|  |  |  |  |  |  |  |  |  |
|  |  |  |  |  |  |  |  |  |
|  |  |  |  |  |  |  |  |  |

Abbreviations:

CI, confidence interval; cnt, control; int, intervention; IRR, incidence rate ratio; mth, month; No., number; NS, not specified; RR, relative risk

BSI, bloodstream infection; C.diff, clostridium difficile infection; CTZ-R, ceftazidime-resistant; CIP-R, ciprofloxacin-resistant; CRE, carbapenem-resistant enterobacteriaceae; GNB, Gram-negative bacteria; HAI, health care-associated infection; KPC, klebsiella pneumoniae carbapenemase-producing K. pneumoniae; MRSA, methicillin-resistant Staphylococcus aureus; VRE, vancomycin-resistant enterococci

CLC, Community living Centers; EH, Elderly home; LTCF, long-term care facility not specified; NH, nursing home

A, axillary; B, blood; C; suprapubic catheter site; N, nasal; P, pharyngeal or throat; I, inguinal or groin; F, enteral feeding tube insertion site; G, gastric fluid; R, rectal or perineal; S, stool or faecal; Sp, sputum; U, urine, V, vaginal; W, wound; Oth, other sites (depending on clinical picture); Unk, unknown

ABHR, alcohol-based hand rub; CAUTI, catheter-associated urinary tract infection; CDC, Centers for Disease Control and Prevention; HH, hand hygiene; ID, infectious disease; HCP, health care personnel; HCW, healthcare workers; SSTI, skin and soft-tissue infection; UTI, urinary tract infection

**References**

1 [Bradley SF, Terpenning MS, Ramsey MA, *et al.* Methicillin-resistant Staphylococcus aureus: colonization and infection in a long-term care facility. *Ann Intern Med* 1991;**115**:417–22. doi:](http://paperpile.com/b/ivsOrE/OIRa)[10.7326/0003-4819-115-6-417](http://dx.doi.org/10.7326/0003-4819-115-6-417)

1. Number of facilities included in the baseline analysis. [↑](#footnote-ref-1)
2. IPC training consisted of a 36-hour course and periodic workshops in infection control principles specific to the LTCF to designated infection control personnel. [↑](#footnote-ref-2)
3. Sample size was not specified. We reported the number of beds in the nursing homes. [↑](#footnote-ref-3)
4. Designated areas included the nurses’ station, at each resident’s bedside, at napkin-round trolley, along the corridor and in the common room. [↑](#footnote-ref-4)
5. The authors reported the mean prevalence. [↑](#footnote-ref-5)
6. WHO multimodal strategies includes (1) supply with alcohol-based hand rub; (2) place alcohol hand rub at points of care; (3) display posters and reminders depicting hand hygiene indications and proper techniques, (4) deliver health talk by trained infectious control branch nurses to HCWs on the rationale, indications, and techniques for HH, methods to prevent skin dryness, and indications for glove use, with its limitations; (5) play video clips tailor-made to HCWs’ working environment (HH opportunities); (6) measure the skin moisture level with a moisture checker before and after application of alcohol-based hand rub to minimize the fear of skin dryness; (7) use a hand inspection cabinet and fluorescent dye as training aids to demonstrate the importance of proper HH techniques; (8) employ the train-the-trainer approach and provide training materials to each intervention home; (9) provide immediate feedback to HCW at the time of direct observation; (10) provide performance reports with anonymous and aggregated data to the management after each phase of data collection. [↑](#footnote-ref-6)
7. Direct observation of HH practice of HCWs in intervention homes were conducted at the baseline and one and four months after intervention by eight trained infection control nurses. [↑](#footnote-ref-7)
8. Hand hygiene compliance was the percentage of a comparison between the number of times hand decontamination occurred versus the number of times hand washing opportunities arising. [↑](#footnote-ref-8)
9. We only included the participants in survey one and four since insufficient information was provided in survey two and three. [↑](#footnote-ref-9)
10. The MRSA prevalence was not reported in numbers. The reported numbers were estimated based on visual assessment from Figure 1 in the paper. [↑](#footnote-ref-10)
11. MRSA infections were defined by the criteria set forth by the Centers for Disease Control. Events were reported with different length of time periods compared with pre-intervention. [↑](#footnote-ref-11)
12. The pre-intervention rate of MRSA infection was extracted from the previous study from the same research team [**[1]**](https://paperpile.com/c/ivsOrE/OIRa). [↑](#footnote-ref-12)
13. The authors defined passive surveillance as cultures collected at baseline, day 15, and then monthly for outcome measurements monthly, with no reports given to the facilities; while active surveillance was defined as cultures collected at baseline, day 15, and then monthly, with data reported back to the facilities every month. [↑](#footnote-ref-13)
14. The denominator (i.e. total number of swabs) were not provided in the article. It was calculated based on the number of positive swabs divided by the total number of swabs. [↑](#footnote-ref-14)
15. Prevalence density rate (cluster- and covariate-adjusted rate ratio) defined as each participant’s total number of MDRO-positive anatomic sites across all MDROs per visit averaged over the duration of participation. [↑](#footnote-ref-15)
16. Risk of new MDRO acquisition defined as the number of residents with new acquisitions per 1000 device-days at risk. [↑](#footnote-ref-16)
17. The data was observed visually from Figure 4. [↑](#footnote-ref-17)
18. The authors used self-reported questionnaires to monitor the staff compliance before and after the interventions implemented [↑](#footnote-ref-18)
19. There was too much resident and staff intermingling between all units each day, and thus the decision was made to make all units intervention sites for Year 2 in order to test if the original intervention plan would have a significant impact. [↑](#footnote-ref-19)
20. Peterson (2016) and Schora (2014) are originated from the same study. The infection rates were reported in Peterson (2016) but not in Schora (2014). [↑](#footnote-ref-20)
21. Schora et al. (2014) and Peterson et al. (2016) are based on the same study with the same study sample. [↑](#footnote-ref-21)
22. No information on randomization on LTCFs [↑](#footnote-ref-22)
23. Existing infection control practices at each LTCF include regular surveillance and reporting, contact isolation for residents with signs and symptoms of an infection caused by a multidrug-resistant organism or unexplained symptoms, and cleaning with bleach on all high-touch areas during outbreak. [↑](#footnote-ref-23)
24. All units followed intervention protocol because of the failure of the cluster randomized approach to sufficiently segregate patients in year 2. [↑](#footnote-ref-24)
25. One LTCF continued with mupirocin decolonization; however, two others replaced mupirocin with retapamulin because of increasing mupirocin resistance in the MRSA isolates of the residents at these two LTCFs. Residents who failed 2 rounds of retapamulin decolonization were offered decolonization with oral rifampin and minocycline. [↑](#footnote-ref-25)
26. All units received intervention due to randomization contamination. [↑](#footnote-ref-26)
27. The monthly hand hygiene compliance monitoring using the hand hygiene observation tool was conducted by the Director of Nursing and the infection preventionist. Special care was taken to perform the monitoring when HCP were unaware of the Director of Nursing and the infection preventionist’s presence in the resident care area to avoid the Hawthorne effect. [↑](#footnote-ref-27)
28. Resident compliance was encouraged and observed but not reported. [↑](#footnote-ref-28)
29. Prevalence was calculated as the number of residents cultured positive who have not already been identified on admission divided by the total number of residents tested after initial screening and decolonization. [↑](#footnote-ref-29)
30. Clinically confirmed LTCF-onset CDI cases was defined as residents with clinical evidence of illness (ie, diarrhea or histopathologic or colonoscopic evidence of pseudomembranous colitis) and a non duplicate, nonrecurrent positive diagnostic laboratory test collected >48 hours after admission [↑](#footnote-ref-30)
