# Supplementary figures and images for: Approaches to multidrug-resistant organism prevention and control in long-term care facilities for older people: a systematic review and meta-analysis
Source: Antimicrob Resist Infect Control. 2022 Jan 15;11:7. doi: 10.1186/s13756-021-01044-0 (PMC8761316; doi:10.1186/s13756-021-01044-0)

**Additional file 5. Forest plot for studies evaluating MRSA colonization.**

**
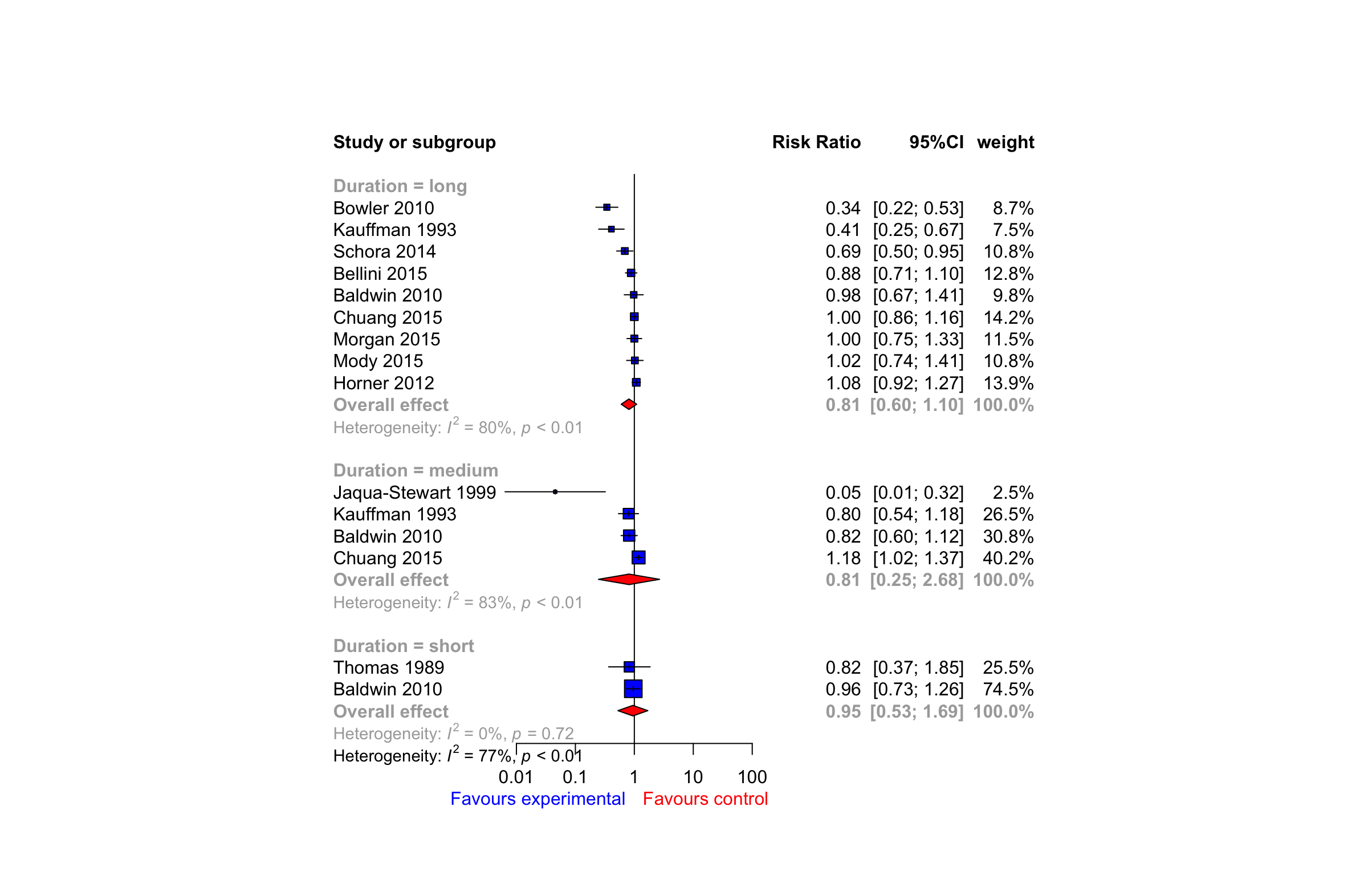
**

Supplement: Supplementary file 5 — Additional file 5. Forest plot for studies evaluating MRSA colonization. [file 13756_2021_1044_MOESM5_ESM.docx]

**Additional file 6. Forest plot for sensitivity analysis.**


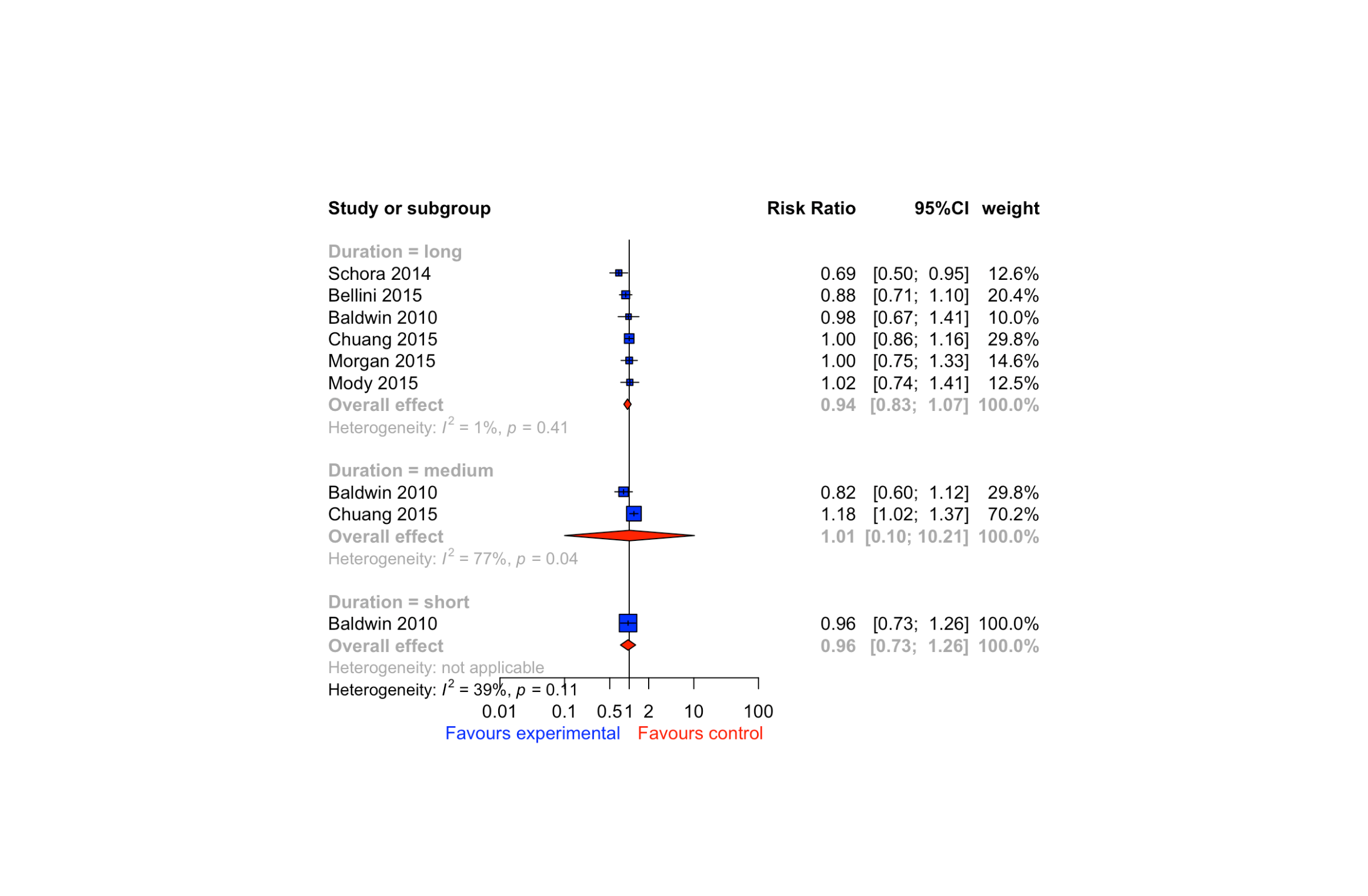

Supplement: Supplementary file 6 — Additional file 6. Forest plot for sensitivity analysis. [file 13756_2021_1044_MOESM6_ESM.docx]
