# Supplementary material for: Approaches to multidrug-resistant organism prevention and control in long-term care facilities for older people: a systematic review and meta-analysis
Source: Antimicrob Resist Infect Control. 2022 Jan 15;11:7. doi: 10.1186/s13756-021-01044-0 (PMC8761316; doi:10.1186/s13756-021-01044-0)
Supplement: Supplementary file 7 — Additional file 7. Risk of bias assessment for randomized trials using Cochrane risk-of-bias tool. [file 13756_2021_1044_MOESM7_ESM.docx]

**Additional file 7.** **Risk of bias assessment for randomized trials using Cochrane risk-of-bias tool.**

Responses underlined in green are potential markers for low risk of bias, and responses in red are potential markers for a risk of bias. Where questions relate only to sign posts to other questions, no formatting is used.

| **Signalling questions** | **Baldwin 2010** | **Bellini 2015** | **Chuang 2015** | **Héquet 2017** | **Ho 2012** | **Mody 2019** | **Schora 2016** | **Peterson 2016** |
| --- | --- | --- | --- | --- | --- | --- | --- | --- |
| Domain 1: Risk of bias arising from the randomization process | | | | | | | | |
| 1.1. Was the allocation sequence random? | Y^^[[1]](#footnote-1)^^ | Y^^[[2]](#footnote-2)^^ | Y^^[[3]](#footnote-3)^^ | Y^^[[4]](#footnote-4)^^ | Y^^[[5]](#footnote-5)^^ | Y^^[[6]](#footnote-6)^^ | NI | NI |
| 1.2. Was the allocation sequence concealed until participants were enrolled and assigned to interventions? | Y^^[[7]](#footnote-7)^^ | Y^^[[8]](#footnote-8)^^ | Y^^[[9]](#footnote-9)^^ | Y^^[[10]](#footnote-10)^^ | Y^^[[11]](#footnote-11)^^ | Y^^[[12]](#footnote-12)^^ | Y^^[[13]](#footnote-13)^^ | Y^^[[14]](#footnote-14)^^ |
| 1.3. Were there baseline imbalances that suggest a problem with the randomization process? | N^^[[15]](#footnote-15)^^ | N^^[[16]](#footnote-16)^^ | N^^[[17]](#footnote-17)^^ | Y^^[[18]](#footnote-18)^^ | N^^[[19]](#footnote-19)^^ | N^^[[20]](#footnote-20)^^ | NI | NI |
| **Risk of bias judgement** | **Low** | **Low** | **Low** | **Some concerns** | **Low** | **Low** | **Low** | **Low** |
|  |  |  |  |  |  |  |  |  |
| Domain 2: Risk of bias due to deviations from the intended interventions (effect of assignment to intervention) | | | | | | | | |
| 2.1. Were participants aware of their assigned intervention during the trial? | PN | PN | PN | PN | N | N | PN | PN |
| 2.2. Were carers and trial personnel aware of participants’ assigned intervention during the trials? | PY | PY | PY | PY | PY | Y | PY | PY |
| 2.3. If Y/PY/NI to 2.1 or 2.2: Were there deviations from the intended intervention that arose because of the trial context? | NI | N^^[[21]](#footnote-21)^^ | NI | NI | NI | N^^[[22]](#footnote-22)^^ | Y^^[[23]](#footnote-23)^^ | Y^^[[24]](#footnote-24)^^ |
| 2.4. If Y/PY to 2.3: Were these deviations from intended intervention unbalanced between groups and likely to have affected the outcome? | NA | NA | NA | NA | NA | NA | Y^^[[25]](#footnote-25)^^ | Y^^[[26]](#footnote-26)^^ |
| 2.5. If Y/PY/NI to 2.4: Were these deviations from intended intervention balanced between groups? | NA | NA | NA | NA | NA | NA | N^^[[27]](#footnote-27)^^ | N^^[[28]](#footnote-28)^^ |
| 2.6. Was an appropriate analysis used to estimate the effect of assignment to intervention? | Y^^[[29]](#footnote-29)^^ | N^^[[30]](#footnote-30)^^ | N^^[[31]](#footnote-31)^^ | PN^^[[32]](#footnote-32)^^ | PN^^[[33]](#footnote-33)^^ | N^^[[34]](#footnote-34)^^ | N^^[[35]](#footnote-35)^^ | NI^^[[36]](#footnote-36)^^ |
| 2.7. If N/PN/NI to 2.6: Was there potential for a substantial impact (on the estimated effect of intervention) of analysing participants in the wrong group? | NA | N^^[[37]](#footnote-37)^^ | N^^[[38]](#footnote-38)^^ | NI | N^^[[39]](#footnote-39)^^ | N^^[[40]](#footnote-40)^^ | NI | PN^^[[41]](#footnote-41)^^ |
| **Risk-of-bias judgement** | **Low** | **Some concerns** | **High** | **High** | **Some concerns** | **Some concerns** | **High** | **High** |
|  |  |  |  |  |  |  |  |  |
| Domain 2: Risk of bias due to deviations from the intended interventions (effect of starting and adhering to intervention) | | | | | | | | |
| 2.1. Were participants aware of their assigned intervention during the trial? | PN^^[[42]](#footnote-42)^^ | PN^^[[43]](#footnote-43)^^ | PN^^[[44]](#footnote-44)^^ | PN^^[[45]](#footnote-45)^^ | N^^[[46]](#footnote-46)^^ | N^^[[47]](#footnote-47)^^ | PN^^[[48]](#footnote-48)^^ | PN^^[[49]](#footnote-49)^^ |
| 2.2. Were carers and people delivering the interventions aware of participants’ assigned intervention during the trials? | PY | PY | PY | PY | PY | Y | PY | PY |
| 2.3. [If applicable:] If Y/PY/NI to 2.1 or 2.2: Were important non-  protocol interventions balanced across intervention groups? | NI^^[[50]](#footnote-50)^^ | PY^^[[51]](#footnote-51)^^ | NI^^[[52]](#footnote-52)^^ | NI^^[[53]](#footnote-53)^^ | NI^^[[54]](#footnote-54)^^ | PY^^[[55]](#footnote-55)^^ | PN^^[[56]](#footnote-56)^^ | PN^^[[57]](#footnote-57)^^ |
| 2.4. [If applicable:] Were there failures in implementing the intervention that could have affected the outcome? | PY^^[[58]](#footnote-58)^^ | PN | PN | PN | PN | PN | Y^^[[59]](#footnote-59)^^ | Y^^[[60]](#footnote-60)^^ |
| 2.5. [If applicable:] Was there non-adherence to the assigned intervention regimen that could have affected participants’ outcomes? | PY^^[[61]](#footnote-61)^^ | PN^^[[62]](#footnote-62)^^ | PY^^[[63]](#footnote-63)^^ | PN^^[[64]](#footnote-64)^^ | PY^^[[65]](#footnote-65)^^ | PY^^[[66]](#footnote-66)^^ | PY^^[[67]](#footnote-67)^^ | PY^^[[68]](#footnote-68)^^ |
| 2.6. If N/PN/NI to 2.3, or Y/PY/NI to 2.4 or 2.5: Was an appropriate analysis used to estimate the effect of adhering to the intervention? | PN^^[[69]](#footnote-69)^^ | NA | N^^[[70]](#footnote-70)^^ | NA | PY^^[[71]](#footnote-71)^^ | N | PN | PN |
| **Risk-of-bias judgement** | **High** | **Low** | **High** | **Low** | **Some concerns** | **High** | **High** | **High** |
|  |  |  |  |  |  |  |  |  |
| Domain 3: Missing outcome data |  |  |  |  |  |  |  |  |
| 3.1. Were data for this outcome available for all, or nearly all, participants randomized? | N^^[[72]](#footnote-72)^^ | N^^[[73]](#footnote-73)^^ | N^^[[74]](#footnote-74)^^ | NI^^[[75]](#footnote-75)^^ | Ys^^[[76]](#footnote-76)^^ | N^^[[77]](#footnote-77)^^ | NI^^[[78]](#footnote-78)^^ | Y^^[[79]](#footnote-79)^^ |
| 3.2. If N/PN/NI to 3.1: Is there evidence that the result was not biased by missing outcome data? | PN | PN | PN | PN | NA | PN | PN | NA |
| 3.3. If N/PN to 3.2: Could missingness in the outcome depend on its true value? | PY | PY | PY | PY | NA | PY | PY | NA |
| 3.4. If Y/PY/NI to 3.3: Is it likely that missingness in the outcome depended on its true value? | PN^^[[80]](#footnote-80)^^ | PY^^[[81]](#footnote-81)^^ | PN^^[[82]](#footnote-82)^^ | NI^^[[83]](#footnote-83)^^ | NA | PN^^[[84]](#footnote-84)^^ | NI^^[[85]](#footnote-85)^^ | NA |
| **Risk-of-bias judgement** | **Some concerns** | **High** | **Some concerns** | **High** | **Low** | **Some concerns** | **High** | **Low** |
|  |  |  |  |  |  |  |  |  |
| Domain 4: Risk of bias in measurement of the outcome |  |  |  |  |  |  |  |  |
| 4.1. Was the method of measuring the outcome inappropriate? | N | N | N | N | N | N | N | N |
| 4.2. Could measurement or ascertainment of the outcome have differed between intervention groups? | PN | PN | PN | PN | PN | PN | PN | PN |
| 4.3. If N/PN/NI to 4.1 and 4.2: Were outcome assessors aware of the intervention received by study participants? | PN | PN | PN | PN | PN | PN | PN | PN |
| 4.4. If Y/PY/NI to 4.3: Could assessment of the outcome have been influenced by knowledge of intervention received? | PN | PN | PN | PN | PN | PN | PN | PN |
| 4.5. If Y/PY/NI to 4.4: Is it likely that assessment of the outcome was influenced by knowledge of intervention received? | NA | NA | NA | NA | NA | NA | NA | NA |
| **Risk-of-bias judgement** | **Low** | **Low** | **Low** | **Low** | **Low** | **Low** | **Low** | **Low** |
|  |  |  |  |  |  |  |  |  |
| Domain 5: Risk of bias in selection of the reported result |  |  |  |  |  |  |  |  |
| 5.1. Were the data that produced this result analysed in accordance with a pre-specified analysis plan that was finalized before unblinded outcome data were available for analysis? | PY | Y | PY | PY | PY | Y | Y | Y |
| Is the numerical result being assessed likely to have been selected, on the basis of the results, from  5.2. … multiple eligible outcome measurements (e.g. scales, definitions, time points) within the outcome domain? | PN | PN | PN | PN | PN | PN | Y^^[[86]](#footnote-86)^^ | Y^^[[87]](#footnote-87)^^ |
| 5.3. … multiple eligible analyses of the data | PN | PN | PN | PN | PN | PN | PN | PN |
| **Risk-of-bias judgement** | **Low** | **Low** | **Low** | **Low** | **Low** | **Low** | **High** | **High** |
|  |  |  |  |  |  |  |  |  |
| Overall risk of bias |  |  |  |  |  |  |  |  |
| **Risk-of-bias judgement** | **High** | **High** | **High** | **High** | **Some concerns** | **High** | **High** | **High** |

**NA, Not applicable; Y, Yes; PY, Possible yes; PN, Possible No; N, No; NI, No information**

Abbreviation:

MRSA, methicillin-resistant Staphylococcus aureus; NH, nursing home; RCHE, residential Care Homes for the elderly; RCT, randomized controlled trial;

1. Done. Since the study is a matched clustered RCT, randomization is done at nursing home level using computer-generated randomization sequence. The process of selecting the blocks was clearly specified. [↑](#footnote-ref-1)
2. Done. Since the study is a clustered RCT, randomization is done at nursing home level using computer-generated randomization sequence. [↑](#footnote-ref-2)
3. Done. Care homes were used as units of randomization. RCHE were randomly allocated to either intervention or control arm. The authors applied stratified block randomization where the stratum was the operation mode (run by non-governmental-organizations or run by the private section) with a block size of two. [↑](#footnote-ref-3)
4. Done. Since the study is a clustered RCT, randomization is done at nursing home level. The technique used in randomization was not specified. [↑](#footnote-ref-4)
5. Done. Since the study is clustered RCT, homes for the elderly were used as units of randomization using random number generator. [↑](#footnote-ref-5)
6. Done. Since the study is clustered RCT, nursing homes were used as unit of randomization using computer-generated randomization sequence. [↑](#footnote-ref-6)
7. Randomization was done by centrally administered randomization. Cluster-randomized trials often randomize all clusters at once, so lack of concealment of an allocation sequence should not usually be an issue. Randomization is done at nursing home level using computer-generated randomization sequence. [↑](#footnote-ref-7)
8. Randomization was done by centrally administered randomization. [↑](#footnote-ref-8)
9. Randomization was done by centrally administered randomization. The randomization list was generated using the “rand” command in Microsoft Excel 2003. [↑](#footnote-ref-9)
10. Randomization was done by centrally administered randomization at nursing home level. [↑](#footnote-ref-10)
11. Randomization was done by centrally administered randomization at nursing home level. [↑](#footnote-ref-11)
12. Randomization was done by centrally administered randomization at nursing home level. [↑](#footnote-ref-12)
13. Randomization was done by centrally administered randomization at nursing home level. [↑](#footnote-ref-13)
14. Randomization was done by centrally administered randomization at nursing home level. [↑](#footnote-ref-14)
15. The baseline characteristics (homes and residents) were largely similar in the intervention and control groups. At baseline, resident MRSA prevalence was comparable in both groups. [↑](#footnote-ref-15)
16. There were no significant differences in baseline characteristics between groups [↑](#footnote-ref-16)
17. There were no significant differences in baseline characteristics between groups [↑](#footnote-ref-17)
18. A significant difference on baseline MRSA prevalence between groups was noted (8.1% vs. 4.0%; P=0.01). [↑](#footnote-ref-18)
19. There were no significant differences in baseline characteristics between groups. Baseline demographics were similar among the 3 arms. [↑](#footnote-ref-19)
20. There were no significant differences in baseline characteristics between groups. [↑](#footnote-ref-20)
21. Trial protocol is available. The interventions are consistent with the trial protocol. [↑](#footnote-ref-21)
22. Trial protocol is available. The interventions are consistent with the trial protocol. [↑](#footnote-ref-22)
23. In year 2, all residents were converted to intervention groups since the study samples were cross-contaminated. The trial context failed to implement the protocol intervention. [↑](#footnote-ref-23)
24. In year 2, all residents were converted to intervention groups since the study samples were cross-contaminated. The trial context failed to implement the protocol intervention. [↑](#footnote-ref-24)
25. The changes from assigned intervention that are inconsistent with the trial protocol and arose because of the trial context. [↑](#footnote-ref-25)
26. The changes from assigned intervention that are inconsistent with the trial protocol and arose because of the trial context. [↑](#footnote-ref-26)
27. The changes from assigned intervention affected the outcome estimates of the control group more than the intervention group since the control group was converted to intervention group due to cross-contamination. [↑](#footnote-ref-27)
28. The changes from assigned intervention affected the outcome estimates of the control group more than the intervention group since the control group was converted to intervention group due to cross-contamination. [↑](#footnote-ref-28)
29. Data was analyzed on an “intention-to-treat analysis”. [↑](#footnote-ref-29)
30. Residents should be analyzed in the groups to which they were randomized, regardless of the intervention that they received. Since the study adopted "post-hoc/ per-protocol analysis", the effect estimates may be biased. [↑](#footnote-ref-30)
31. Although the authors stated the analysis was undertaken on an intention-to-treat basis. However, the authors analyzed the data after excluding the residents who died and moved out from the facilities. [↑](#footnote-ref-31)
32. Probably no. The authors did not report the number randomized; we have insufficient reporting of attrition/ exclusions to permit judgement. Since the study is an extension study of Bellini (2015)[[2]](https://paperpile.com/c/ivsOrE/YP8Z) which adopted “post-hoc/per-protocol analysis”, we assumed the appropriate analysis was probably not done. [↑](#footnote-ref-32)
33. The authors did not report any analysis used to estimate the effect of assignment to intervention. [↑](#footnote-ref-33)
34. The authors analyzed the data after excluding the residents lost in follow-up (per-protocol analysis). [↑](#footnote-ref-34)
35. The authors evaluated the differences between time periods using the fisher exact test for proportion. No analytical methods used to estimate the effect of assignment to intervention was used. [↑](#footnote-ref-35)
36. Insufficient reporting of analytical methods used to estimate the effect of assignment to intervention. The authors stated that less than 1% of the patients or their families in this ‘Op-Out design’ declined participation. [↑](#footnote-ref-36)
37. One NH in the intervention group was excluded for final analysis. There may not be any potential for substantial impact since fewer than 2% of NHs were excluded in the final analysis. [↑](#footnote-ref-37)
38. The turnover rate was 33.1% and 30.1% in the intervention group and control group respectively. The number excluded from the analysis was balanced between groups. There should not have been a substantial impact on the result. [↑](#footnote-ref-38)
39. There was no loss to follow-up. The effect of analysis used to estimate the effect of assignment to intervention should be minimal. [↑](#footnote-ref-39)
40. 49 out of 203 residents and 53 out of 215 residents in the intervention and control group were excluded in the analysis respectively. The number excluded from the analysis was balanced between groups. There should not have been a substantial impact on the result. [↑](#footnote-ref-40)
41. Since less than 1% of the patients or their families in this ‘Op-Out design’ declined participation. There should not have been a substantial impact on the result. [↑](#footnote-ref-41)
42. Participants probably were unaware of assigned groups during the trial since the randomization was done at facility level. But any deviation from intended intervention was unlikely to impact on the outcome. [↑](#footnote-ref-42)
43. Participants probably were unaware of assigned groups during the trial since the randomization was done at facility level. But any deviation from intended intervention was unlikely to impact on the outcome. [↑](#footnote-ref-43)
44. Participants probably were unaware of assigned groups during the trial since the randomization was done at facility level. But any deviation from intended intervention was unlikely to impact on the outcome. [↑](#footnote-ref-44)
45. Participants probably were unaware of assigned groups during the trial since the randomization was done at facility level. But any deviation from intended intervention was unlikely to impact on the outcome. [↑](#footnote-ref-45)
46. Participants were blinded to the allocation to intervention or control arms. [↑](#footnote-ref-46)
47. Participants were blinded to the allocation to intervention or control arms. [↑](#footnote-ref-47)
48. Participants probably were unaware of their assigned groups during the trial since the randomization was done at facility level. But any deviation from intended intervention was unlikely to impact on the outcome. [↑](#footnote-ref-48)
49. Participants probably were unaware of their assigned groups during the trial since the randomization was done at facility level. But any deviation from intended intervention was unlikely to impact on the outcome. [↑](#footnote-ref-49)
50. Study protocol is not available. Trialists did not report whether deviations arose because of the trial context. [↑](#footnote-ref-50)
51. Study protocol is available. No non-protocol interventions were observed. [↑](#footnote-ref-51)
52. Study protocol is not available. Trialists did not report whether deviations arose because of the trial context. [↑](#footnote-ref-52)
53. Study protocol is not available. Trialists did not report whether deviations arose because of the trial context. [↑](#footnote-ref-53)
54. Study protocol is not available. Trialists did not report whether deviations arose because of the trial context. [↑](#footnote-ref-54)
55. Study protocol is available. No non-protocol interventions were observed. [↑](#footnote-ref-55)
56. Study protocol is available. Education and environmental cleaning were not described in the trial protocol in the intervention arm. [↑](#footnote-ref-56)
57. Study protocol is available. Education and environmental cleaning were not described in the trial protocol in the intervention arm. [↑](#footnote-ref-57)
58. The authors reported failure in engaging the management in the intervention. Despite regular feedback from the infection control nurse to the home managers on poor audit findings, there were limited attempts by management to address non-compliance over the study period. [↑](#footnote-ref-58)
59. In year 2, all residents were converted to intervention groups since the study samples were cross-contaminated. The trial context failed to implement the protocol interventions. The failure in implementing the intervention could have affected the outcome. [↑](#footnote-ref-59)
60. In year 2, all residents were converted to intervention groups since the study samples were cross-contaminated. The trial context failed to implement the protocol interventions. The failure in implementing the intervention could have affected the outcome. [↑](#footnote-ref-60)
61. The authors reported failure in engaging the management in the intervention. Despite regular feedback from the infection control nurse to the home managers on poor audit findings, there were limited attempts by management to address non-compliance over the study period. The compliance of hand hygiene was 66% and decontamination of equipment was 75% in the intervention group. [↑](#footnote-ref-61)
62. Interventions were implemented in the form of training and decolonization. The risk of imperfect adherence was minimal. [↑](#footnote-ref-62)
63. The Hand hygiene compliance among RCHE staff was only 45.6% and 7.2% in the intervention and control group, respectively. [↑](#footnote-ref-63)
64. Interventions were implemented in the form of training and decolonization. The risk of imperfect adherence was minimal. [↑](#footnote-ref-64)
65. The Hand hygiene compliance among RCHE staff were only 21.5%, 60.6% and 48.6% in control group, intervention group 1 and intervention group 2, respectively. [↑](#footnote-ref-65)
66. The Hand hygiene and gown use compliance among RCHE staff were 37.3% and 40.5% in the intervention group while that were 18.2% and 1.8% in control group, respectively. [↑](#footnote-ref-66)
67. Interventions were implemented in the form of education, decolonization and environmental cleaning. The risk of imperfect adherence was minimal. [↑](#footnote-ref-67)
68. Interventions were implemented in the form of education, decolonization and environmental cleaning. The risk of imperfect adherence was minimal. [↑](#footnote-ref-68)
69. The authors used paired sample t-test in analyzing the data. It is not able to derive an unbiased estimate of the effect of adhering to intervention. [↑](#footnote-ref-69)
70. Although the authors stated the analysis was undertaken on an intention-to-treat basis i.e., residents should be analysed in the groups to which they were randomized, regardless of the intervention that they received. However, the authors analyzed the data after excluding the residents who died and moved out from the facilities. [↑](#footnote-ref-70)
71. The authors did not report any analysis used to estimate the effect of assignment to intervention. However, there was no loss to follow-up of these 18 homes. The effect of analysis used to estimate the effect of adhering to the intervention should be minimal. [↑](#footnote-ref-71)
72. The total residents lost to follow-up in the intervention group was 158 (40% of total residents in the intervention group) and in the control group was 157 (39% of total residents in the control group). [↑](#footnote-ref-72)
73. The authors stated that the proportion of residents who accepted and underwent MRSA screening was heterogeneous, ranging from 27% to 100% (mean 86%) in control NHs and from 20% to 100% (mean, 87%) in intervention NHs. [↑](#footnote-ref-73)
74. The turnover rate was 33.1% in the intervention group while that was 30.1% in the control group. The number of participants with missing outcome data is sufficiently large that their outcomes could have made an important difference to the estimated effect of intervention. [↑](#footnote-ref-74)
75. The authors stated that all residents who gave verbal informed consent were screened for MRSA carriage. The exact number of screenings was not described. [↑](#footnote-ref-75)
76. The authors reported there was no loss to follow-up of the 18 homes. [↑](#footnote-ref-76)
77. 203 nursing home residents were enrolled into the intervention group while 215 were enrolled into the control group. Only 154 and 162 residents with more than 1 follow-up in the intervention and control group respectively. The authors excluded residents without only a baseline visit and no follow-up visit from analysis. The number of participants with missing outcome data is sufficiently large that their outcomes could have made an important difference to the estimated effect of intervention. [↑](#footnote-ref-77)
78. The authors did not report the missingness on the outcome data. [↑](#footnote-ref-78)
79. The authors stated that less than 1% of the patients or their families in this ‘Op-Out design’ declined participation. [↑](#footnote-ref-79)
80. The analysis accounted for participant characteristics that are likely to explain the relationship between missingness in the outcome and its true value. The difference between intervention groups in the proportions of missing outcome data is minimal (158 vs. 157). Reported reasons for missing outcome data seems to be similar between groups. [↑](#footnote-ref-80)
81. The significant proportion of unidentified MRSA carriers may affect the MRSA transmission to different extent between groups. Even though the proportion of screening was balanced between groups, the reasons for rejection were not reported by the authors. If the reasons for rejection were different in the intervention and control group, the risk of baseline imbalance may present which may cause bias in the intervention effect estimates. [↑](#footnote-ref-81)
82. The analysis accounted for participant characteristics that are likely to explain the relationship between missingness in the outcome and its true value. The difference between intervention groups in the proportions of missing outcome data is minimal (33.1% vs. 30.1%). Reported reasons for missing outcome data seems to be similar between groups. [↑](#footnote-ref-82)
83. The authors stated that all residents who gave verbal informed consent were screened for MRSA carriage. The exact number of screening and missing in both groups was not described. [↑](#footnote-ref-83)
84. The analysis accounted for participant characteristics that are likely to explain the relationship between missingness in the outcome and its true value. The difference between intervention groups in the proportions of missing outcome data is minimal. Reported reasons for missing outcome data seems to be similar between groups. [↑](#footnote-ref-84)
85. The authors did not provide any information on the differences between intervention groups in the proportions of missing outcome data. [↑](#footnote-ref-85)
86. Study protocol is available. MRSA acquisition rate was not on the pre-specified outcomes. [↑](#footnote-ref-86)
87. Study protocol is available. MRSA infection rate was not on the pre-specified outcomes. [↑](#footnote-ref-87)
