# Supplementary material for: Approaches to multidrug-resistant organism prevention and control in long-term care facilities for older people: a systematic review and meta-analysis
Source: Antimicrob Resist Infect Control. 2022 Jan 15;11:7. doi: 10.1186/s13756-021-01044-0 (PMC8761316; doi:10.1186/s13756-021-01044-0)
Supplement: Supplementary file 8 — Additional file 8. Risk of bias assessment for non-randomized intervention studies using Cochrane risk of bias in non-randomized studies of intervention tool. [file 13756_2021_1044_MOESM8_ESM.docx]

**Additional file 8. Risk of bias assessment for non-randomised interventions studies using risk of bias in non-randomized studies of intervention tool.**

Responses underlined in green are potential markers for low risk of bias, and responses in red are potential markers for a risk of bias. Where questions relate only to sign posts to other questions, no formatting is used.

| **Signalling questions** | **Ben-David 2019** | **Bowler 2010** | **Horner 2012** | **Jaqua-Stewart 1999** | **Kauffman 1993** | **Morgan 2019** | **Ostrowsky 2001** | **Schweon 2013** | **Silverblatt 2000** | **Singh 2018** | **Thomas 1989** |
| --- | --- | --- | --- | --- | --- | --- | --- | --- | --- | --- | --- |
| **Bias due to confounding** | | | | | | | | | | | |
| 1.1 Is there potential for confounding of the effect of intervention in this study? *Selection based on characteristics observed before the start of intervention can be addressed by controlling for imbalanced between experimental intervention and comparator groups in baseline characteristics that are prognostic for the outcome (baseline confounding)  **If N/PN to 1.1:** the study can be considered to be at low risk of bias due to confounding and no further signalling questions need be considered  **If Y/PY to 1.7**: determine whether there is a need to assess time-varying confounding: | Y | Y | PN^^[[1]](#footnote-1)^^ | Y | Y | Y | Y | Y | Y | Y | Y |
| 1.2. Was the analysis based on splitting participants’ follow up time according to intervention received (*partition follow-up time into period)?  **If N/PN**, answer questions relating to baseline confounding (1.4 to 1.6)  **If Y/PY**, go to question 1.3. | N | N | NA | N | N | N | N | N | N | N | N |
| 1.3. Were intervention discontinuations or switches likely to be related to factors that are prognostic for the outcome?  **If N/PN**, answer questions relating to baseline confounding (1.4 to 1.6)  **If Y/PY**, answer questions relating to both baseline and time-varying confounding (1.7 and 1.8) | NA | NA | NA | NA | NA | NA | NA | NA | NA | NA | NA |
| **Questions relating to baseline confounding only** | | | | | | | | |  |  |  |
| 1.4. Did the authors use an appropriate analysis method that controlled for all the important confounding domains? *Did the authors used an appropriate analysis method that accounts for time trends and patterns, and controls for all the important confounding domains? (for before-after studies) | N | N | NA | N | N | Y^^[[2]](#footnote-2)^^ | PN^^[[3]](#footnote-3)^^ | PN^^[[4]](#footnote-4)^^ | PN^^[[5]](#footnote-5)^^ | N | N |
| 1.5. **If Y/PY to 1.4**: Were confounding domains that were controlled for measured validly and reliably by the variables available in this study? | NA | NA | NA | NA | NA | N | NA | NA | NA | NA | NA |
| 1.6. Did the authors control for any post-intervention variables that could have been affected by the intervention? | N | N | NA | N | N | N | N | N | N | N | N |
| *Were measurements of outcomes made at sufficient pre-intervention time points to permit characterisation pre-intervention trends and patterns? (for before-after studies) | PN^^[[6]](#footnote-6)^^ | PN^^[[7]](#footnote-7)^^ | NA | PN^^[[8]](#footnote-8)^^ | Y^^[[9]](#footnote-9)^^ | Y^^[[10]](#footnote-10)^^ | PN^^[[11]](#footnote-11)^^ | PN^^[[12]](#footnote-12)^^ | PN^^[[13]](#footnote-13)^^ | Y^^[[14]](#footnote-14)^^ | N^^[[15]](#footnote-15)^^ |
| *Were there any extraneous events or changes in context around the time of the intervention that could have influenced the outcome? (for before-after studies) | PN | PN | NA | PN | Y^^[[16]](#footnote-16)^^ | PN | PN | PN | PN | PN | PN |
| **Questions relating to baseline and time-varying confounding** | | | | | | | | |  |  |  |
| 1.7. Did the authors use an appropriate analysis method that controlled for all the important confounding domains and for time-varying confounding? | NA | NA | NA | NA | NA | NA | NA | NA | NA | NA | NA |
| 1.8. **If Y/PY to 1.7**: Were confounding domains that were controlled for measured validly and reliably by the variables available in this study? | NA | NA | NA | NA | NA | NA | NA | NA | NA | NA | NA |
| **Risk of bias judgement** | **Serious** | **Serious** | **Low** | **Serious** | **Serious** | **Moderate** | **Moderate** | **Serious** | **Serious** | **Serious** | **Serious** |
|  | | | | | | | | | | | |
| **Bias in selection of participants into the study** | | | | | | | | | | | |
| 2.1. Was selection of participants into the study (or into the analysis) based on participant characteristics observed after the start of intervention?  **If N/PN to 2.1:** go to 2.4 | N | N | N | N | N | N | N | N | N | N | N |
| 2.2. **If Y/PY to 2.1**: Were the post-intervention variables that influenced selection likely to be associated with intervention? | NA | NA | NA | NA | NA | NA | NA | NA | NA | NA | NA |
| 2.3 **If Y/PY to 2.2**: Were the post-intervention variables that influenced selection likely to be influenced by the outcome or a cause of the outcome? | NA | NA | NA | NA | NA | NA | NA | NA | NA | NA | NA |
| 2.4. Do start of follow-up and start of intervention coincide for most participants? (avoid lead time bias) | PY | PY | Y | PY | PY | PY | PY | PY | PY | PY | PY |
| 2.5. **If Y/PY to 2.2 and 2.3, or N/PN to 2.4**: Were adjustment techniques used that are likely to correct for the presence of selection biases? | NA | NA | NA | NA | NA | NA | NA | NA | NA | NA | NA |
| **Risk of bias judgement**  *For studies that prospectively follow a specific group of units from pre-intervention to post-intervention, selection bias is unlikely. For repeated cross-sectional surveys of a population, there is the potential for selection bias even if the study is prospective. (reference:* [*https://training.cochrane.org/handbook/current/chapter-25#_Ref533157666*](https://training.cochrane.org/handbook/current/chapter-25#_Ref533157666)*)* | **Low** | **Low** | **Low** | **Low** | **Low** | **Low** | **Low** | **Low** | **Low** | **Low** | **Low** |
|  | | | | | | | | | | | |
| **Bias in classification of interventions** | | | | | | | | | | | |
| 3.1 Were intervention groups clearly defined? For population-level interventions, the answer to this question is likely to be ‘Yes’. | Y | Y | Y | PY | Y | Y | Y | Y | Y | Y | Y |
| 3.2 Was the information used to define intervention groups recorded at the start of the intervention? *Avoid differential misclassification of intervention status) – For population-level interventions, the answer to this question is likely to be ‘Yes’. | Y | Y | Y | Y | Y | Y | Y | Y | Y | Y | Y |
| 3.3 Could classification of intervention status have been affected by knowledge of the outcome or risk of the outcome? / *Was specification of the distinction between pre-intervention time points and post-intervention time points could have been influenced by the outcome data? (for before-after studies) | N | N | N | N | N | N | N | N | N | N | N |
| **Risk of bias judgement** | **Low** | **Low** | **Low** | **Low** | **Low** | **Low** | **Low** | **Low** | **Low** | **Low** | **Low** |
|  | | | | | | | | | | | |
| **Bias due to deviations from intended interventions** | | | | | | | | | | | |
| **If your aim for this study is to assess the effect of assignment to intervention, answer questions 4.1 and 4.2** | | | | | | | | | | | |
| 4.1. Were there deviations from the intended intervention beyond what would be expected in usual practice? | N | N | N | N | N | N | N | N | N | N | N |
| 4.2. **If Y/PY to 4.1**: Were these deviations from intended intervention unbalanced between groups *and* likely to have affected the outcome? | NA | NA | N | NA | NA | NA | NA | NA | NA | NA | NA |
| *Were the effects of any preparatory (pre-interruption) phases of the intervention were appropriately accounted for? (for before-after studies) | PN | PN | Y | PN | PN | Y^^[[17]](#footnote-17)^^ | PN | PN | PN | Y^^[[18]](#footnote-18)^^ | PN |
| **Risk of bias judgement** | **Moderate** | **Moderate** | **Low** | **Moderate** | **Moderate** | **Low** | **Moderate** | **Moderate** | **Moderate** | **Low** | **Moderate** |
|  | | | | | | | | | | | |
| **Bias due to missing data** | | | | | | | | | | | |
| 5.1 Were outcome data available for all, or nearly all, participants? *Were outcome data missing for whole clusters (units of multiple individuals) as well as for individual participants (for before-after studies)? | PY | N^^[[19]](#footnote-19)^^ | N^^[[20]](#footnote-20)^^ | PY | N^^[[21]](#footnote-21)^^ | PY | PY^^[[22]](#footnote-22)^^ | Y | N^^[[23]](#footnote-23)^^ | PN^^[[24]](#footnote-24)^^ | N^^[[25]](#footnote-25)^^ |
| 5.2 Were participants excluded due to missing data on intervention status? | PN | N | PN | PN | Y^^[[26]](#footnote-26)^^ | PY | Y^^[[27]](#footnote-27)^^ | PN | Y^^[[28]](#footnote-28)^^ | PY^^[[29]](#footnote-29)^^ | PY |
| 5.3 Were participants excluded due to missing data on other variables needed for the analysis? | PN | N | PN | PN | Y^^[[30]](#footnote-30)^^ | PY | PN | PN | PN | PY^^[[31]](#footnote-31)^^ | PY |
| 5.4 **If PN/N to 5.1, or Y/PY to 5.2 or 5.3**: Are the proportion of participants and reasons for missing data similar across interventions? | NA | NI | NI | NA | NI | NI | NA | NA | NA | NI | NI |
| 5.5 **If PN/N to 5.1, or Y/PY to 5.2 or 5.3**: Is there evidence that results were robust to the presence of missing data? | NA | PY | PY^^[[32]](#footnote-32)^^ | NA | PN | PN | PN^^[[33]](#footnote-33)^^ | NA | NA | PN | PN |
| **Risk of bias judgement** | **Low** | **Moderate** | **Moderate** | **Low** | **Serious** | **Serious** | **Moderate** | **Low** | **Moderate** | **Serious** | **Serious** |
|  | | | | | | | | | | | |
| **Bias in measurement of outcomes** | | | | | | | | | | | |
| 6.1 Could the outcome measure have been influenced by knowledge of the intervention received? | N | N | N | N | N | N | N | N | N | N | N |
| 6.2 Were outcome assessors aware of the intervention received by study participants? | PN | PN | N | PN | PN | PN | PN | PN | PN | PN | PN |
| 6.3 Were the methods of outcome assessment comparable across intervention groups? *Were the methods of outcome assessment comparable before and after the intervention (for before-after studies)? | PY | PY | PY | PY | PY | PY | PY | PY | PY | PY | PY |
| 6.4 Were any systematic errors in measurement of the outcome related to intervention received? *Were any changes in systematic errors in measurement of the outcome coincident with implementation of the intervention (for before-after studies)? | PN | PN | PN | PN | PN | PN | PN | PN | PN | PN | PN |
| **Risk of bias judgement** | **Low** | **Low** | **Low** | **Low** | **Low** | **Low** | **Low** | **Low** | **Low** | **Low** | **Low** |
|  | | | | | | | | | | | |
| **Bias in selection of the reported result** | | | | | | | | | | | |
| Is the reported effect estimate likely to be selected, on the basis of the results, from...  7.1. ... multiple outcome *measurements* within the outcome domain? | PN | PN | PN | PN | PN | PN | PN | PN | PN | PN | PN |
| 7.2 ... multiple *analyses* of the intervention-outcome relationship? | PN | PN | PN | PN | PN | PN | PN | PN | PN | PN | PN |
| 7.3 ... different *subgroups*? | PN | PN | PN | PN | PN | PN | PN | PN | PN | PN | PN |
| **Risk of bias judgement** | **Moderate** | **Moderate** | **Moderate** | **Moderate** | **Moderate** | **Moderate** | **Moderate** | **Moderate** | **Moderate** | **Moderate** | **Moderate** |
|  | | | | | | | | | | | |
| **Overall bias** | | | | | | | | | | | |
| **Risk of bias judgement** | **Serious** | **Serious** | **Moderate** | **Serious** | **Serious** | **Serious** | **Serious** | **Serious** | **Serious** | **Serious** | **Serious** |

**NA, Not applicable; Y, Yes; PY, Possible yes; PN, Possible No; N, No; NI, No information**

Abbreviation:

C. diff, Clostridium difficile; HAI; hospital acquired infections; LTCF, long term care facility; MRSA, methicillin-resistant Staphylococcus aureus

* Additional issues or criteria were considered for interrupted time-series studies based on <https://training.cochrane.org/handbook/current/chapter-25#_Ref533157666> (highlighted in blue)

1. Care homes were randomly allocated into three groups. Random allocation was stratified by number of beds and baseline MRSA prevalence. [↑](#footnote-ref-1)
2. The authors include variables that were potential risk factors for confounders based on the literature in the generalized estimating equations models. [↑](#footnote-ref-2)
3. The prevalence rates for each year were compared with the use of a crude chi-square test, Mantel–Haenszel adjusted chi-square test, and a chi-square test for trend. [↑](#footnote-ref-3)
4. The authors adjusted for seasonality only. [↑](#footnote-ref-4)
5. The authors did not control for potential confounders at the analysis stage. [↑](#footnote-ref-5)
6. Only one time point has been measured at the pre-intervention stage. [↑](#footnote-ref-6)
7. Only one time point has been measured at the pre-intervention stage. [↑](#footnote-ref-7)
8. Only two time points have been measured at the pre-intervention stage with large differences in nosocomial infection rate (33% in Oct 1993 and 8.5 for the year of 1993). [↑](#footnote-ref-8)
9. The mean monthly colonization rate for MRSA in all residents was reported prior to the use of mupirocin. [↑](#footnote-ref-9)
10. The quarterly MRSA health care-associated infection rate was reported two years before and after interventions were implemented. [↑](#footnote-ref-10)
11. Only one time point has been measured at the pre-intervention stage. [↑](#footnote-ref-11)
12. Only one time point has been measured at the pre-intervention stage for MRSA infection outcome. [↑](#footnote-ref-12)
13. Only one time point has been measured at the pre-intervention stage. [↑](#footnote-ref-13)
14. The authors reported the clinically confirmed LTCF onset Clostridium difficile infection two years before the intervention was implemented. [↑](#footnote-ref-14)
15. The authors only performed two cross-sectional surveys (pre-intervention and post-intervention surveys). [↑](#footnote-ref-15)
16. From mid-June 1990 to the end of December 1990, patients colonized with MRSA in their anterior nares were treated with nasal application of mupirocin; wounds were not treated. From January 1, 1991, to June 1, 1991, mupirocin was used in both anterior nares and wounds. [↑](#footnote-ref-16)
17. The authors reported the MRSA HAI rate two years before and after intervention was implemented. [↑](#footnote-ref-17)
18. The authors reported the clinically confirmed LTCF onset C.diff. infections two years before and after intervention was implemented. [↑](#footnote-ref-18)
19. 48 out of 147 patients were lost to follow-up due to death and discharge. [↑](#footnote-ref-19)
20. Following the first survey, two homes withdrew from the study leaving 66 homes in the second survey. A further home withdrew following survey 2 leaving 65 homes in surveys 3 and 4. The study analyses report data from those homes that participated in all four surveys. Participation of residents was voluntary, and on average, 46% of the residents were tested for MRSA colonization. Reasons for non-participation of residents were not collected; care homes for people with dementia were not specifically excluded from the study, but residents with dementia were excluded. [↑](#footnote-ref-20)
21. Per-protocol analysis was performed in handling missing cases. There were 24 colonized patients (36%) not included in the analysis of the efficacy of mupirocin due to refusal of treatment/ surveillance, incomplete treatment, discharged from the facility and death. [↑](#footnote-ref-21)
22. The number of cultured to the number of eligible was 90% in 1997 (pre-intervention), 95% in 1998 (post-intervention), and 94% in 1999 (post-intervention), respectively. [↑](#footnote-ref-22)
23. Per-protocol analysis was performed in handling missing cases. There were only 69 of 207 patients (33.3%) cultured at baseline. [↑](#footnote-ref-23)
24. The authors only analyzed data entered into the system. [↑](#footnote-ref-24)
25. Only 75% of the residents in the first survey participated in the second survey. [↑](#footnote-ref-25)
26. Per-protocol analysis was performed in handling missing cases. There were 24 colonized patients (36%) not included in the analysis of the efficacy of mupirocin due to refusal of treatment/ surveillance, incomplete treatment, discharged from the facility and death. [↑](#footnote-ref-26)
27. Per-protocol analysis was performed in handling missing cases. Only data from the 30 facilities that participated in all three years of the study were included. [↑](#footnote-ref-27)
28. Per-protocol analysis was performed in handling missing cases. There were only 69 of 207 patients (33.3%) cultured at baseline. [↑](#footnote-ref-28)
29. The authors only analyzed data entered into the system. [↑](#footnote-ref-29)
30. Per-protocol analysis was performed in handling missing cases. There were 24 colonised patients (36%) not included in the analysis of the efficacy of mupirocin due to refusal of treatment/ surveillance, incomplete treatment, discharged from the facility and death. [↑](#footnote-ref-30)
31. The authors only analysed data entered into the system. [↑](#footnote-ref-31)
32. Per-protocol analysis was performed in handling missing clusters. However, sensitivity analyses were performed for participants present in at least two surveys and those did not. [↑](#footnote-ref-32)
33. Per-protocol analysis was performed in handling missing cases. Only data from the 30 facilities that participated in all three years of the study were included. [↑](#footnote-ref-33)
