# Supplementary material for: Approaches to multidrug-resistant organism prevention and control in long-term care facilities for older people: a systematic review and meta-analysis
Source: Antimicrob Resist Infect Control. 2022 Jan 15;11:7. doi: 10.1186/s13756-021-01044-0 (PMC8761316; doi:10.1186/s13756-021-01044-0)
Supplement: Supplementary file 9 — Additional file 9. Contour-enhanced funnel plot of studies. [file 13756_2021_1044_MOESM9_ESM.docx]

**Additional file 9. Contour-enhanced funnel plot of studies.**


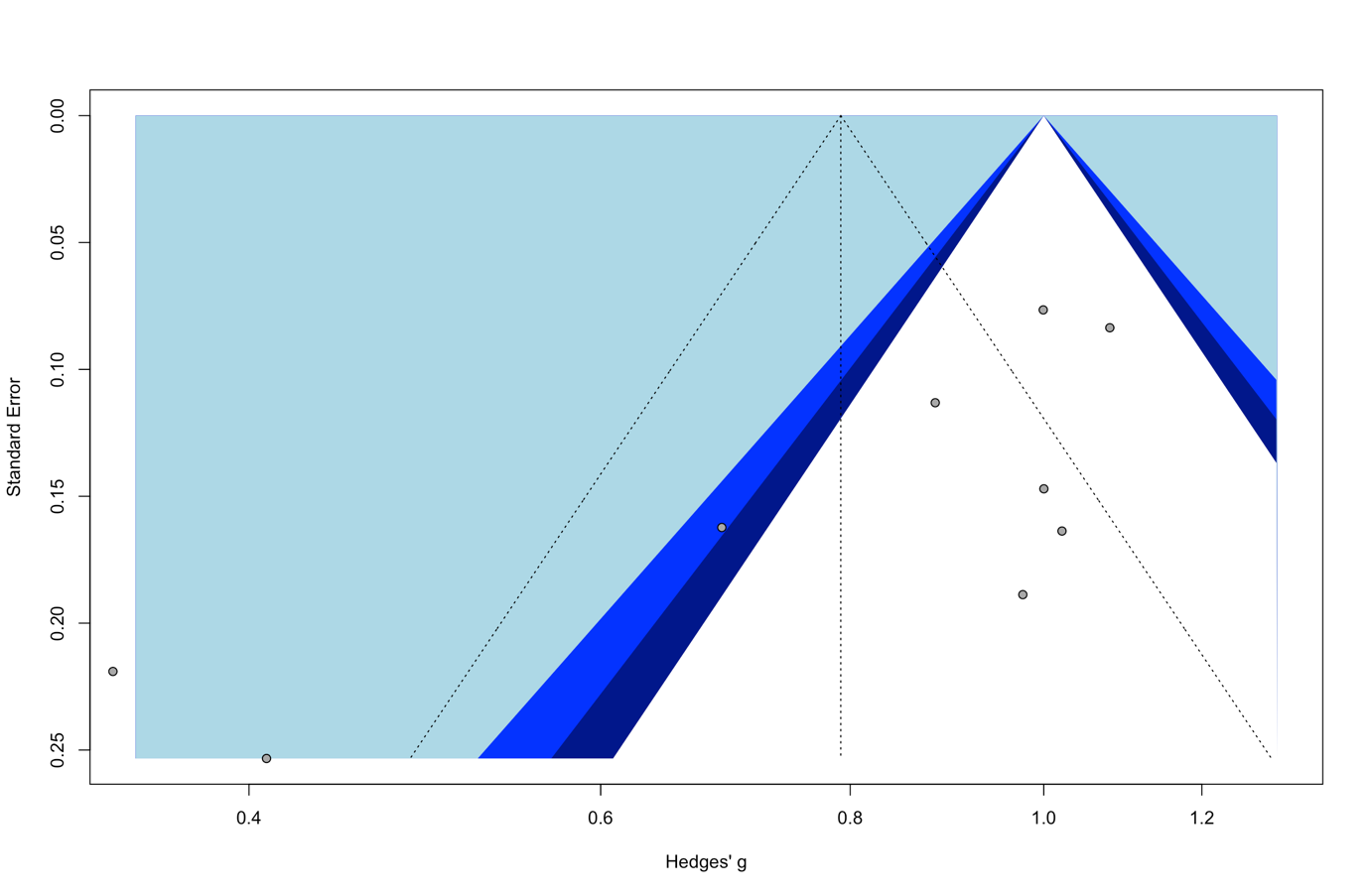


Hedges’g is a measure of effect size. The funnel is centred at one, i.e., at the value under the null hypothesis of no effects. The shaded regions indicate various levels of statistical significance of studies. White region in the middle corresponds to a p-value greater than 0.05; dark blue-shaded to a p-values between 0.025 and 0.05; light blue-shaded to a p-value between 0.01 to 0.025; the region outside the funnel to a p-value below 0.01.
